# MolModa: accessible and secure molecular docking in a web browser

**DOI:** 10.1093/nar/gkae406

**Published:** 2024-05-23

**Authors:** Yuri Kochnev, Mayar Ahmed, Alex M Maldonado, Jacob D Durrant

**Affiliations:** Department of Biological Sciences, University of Pittsburgh, Pittsburgh, PA, USA; Department of Biological Sciences, University of Pittsburgh, Pittsburgh, PA, USA; Department of Biological Sciences, University of Pittsburgh, Pittsburgh, PA, USA; Department of Biological Sciences, University of Pittsburgh, Pittsburgh, PA, USA

## Abstract

Molecular docking advances early-stage drug discovery by predicting the geometries and affinities of small-molecule compounds bound to drug-target receptors, predictions that researchers can leverage in prioritizing drug candidates for experimental testing. Unfortunately, existing docking tools often suffer from poor usability, data security, and maintainability, limiting broader adoption. Additionally, the complexity of the docking process, which requires users to execute a series of specialized steps, often poses a substantial barrier for non-expert users. Here, we introduce MolModa, a secure, accessible environment where users can perform molecular docking entirely in their web browsers. We provide two case studies that illustrate how MolModa provides valuable biological insights. We further compare MolModa to other docking tools to highlight its strengths and limitations. MolModa is available free of charge for academic and commercial use, without login or registration, at https://durrantlab.com/molmoda.

## Graphical Abstract

**Graphical Abstract:**
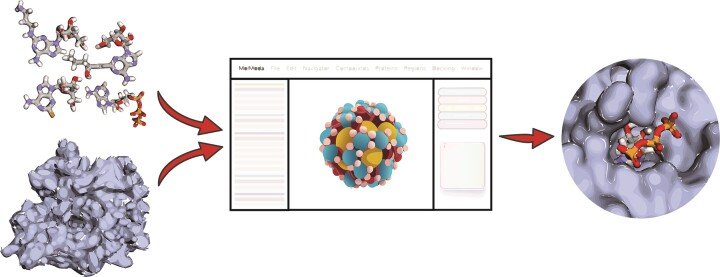

## Introduction

Structure-based computer-aided drug design (CADD) accelerates early-stage drug discovery (1,2) by leveraging experimentally resolved structures to identify drug candidates *in silico*. Molecular docking is a critical CADD technique that predicts the geometries and affinities with which small-molecule ligands (e.g. candidate drugs) might bind to a (protein) target (3–5). Docking involves positioning (*docking*) a small molecule within a protein-target binding pocket and calculating (*scoring*) the geometry of the interaction to assess the likelihood of binding (6). Researchers then prioritize the compounds with the best docking scores for further analysis or experimental testing.

Effective docking requires a series of specialized steps that are difficult for non-experts to execute successfully. Many docking tools only have text-based command-line interfaces (CLIs) and so lack the usability required for broader adoption. Those with graphical user interfaces (GUIs) are often closed-source, expensive, or have restrictive licenses even for academic use (7–9). Cloud-based solutions (i.e. server applications) seek to address these challenges (10–14), but these require users to upload their proprietary data to a third-party server, raising concerns about data security and intellectual property (IP) protection. Further, remote servers rely on consistent funding and staffing to ensure continued availability; if staff members change roles or funding runs out, critical server-based tools may become inaccessible.

Here, we present MolModa, a secure, accessible browser platform where users can perform the multiple steps of a typical molecular-docking workflow. Like a server application, MolModa provides a user-friendly, browser-accessible GUI that does not require users to install software on their computers. But rather than send users’ data to a remote server for analysis, MolModa downloads the analysis software to the users’ browsers and automatically runs the calculations locally, thus ensuring complete IP protection and eliminating the need for difficult-to-maintain remote infrastructure (15). Indeed, hosting the MolModa browser app is no more demanding than hosting a standard website because the heavy calculations run entirely on users’ local computers. MolModa is available free of charge for academic and commercial use, without login or registration, at https://durrantlab.com/molmoda.

## Materials and methods

### MolModa use

MolModa's extensive online documentation (https://durrantlab.com/molmoda/docs/) and accessible sample data (available through *File → Example…*) make it easy for users to familiarize themselves with each step of the docking workflow. The help system also lists all integrated tools alphabetically, with brief descriptions and instructions for access. Here, we provide only a brief overview of MolModa use.

#### Loading molecular structures

**Figure 1.**
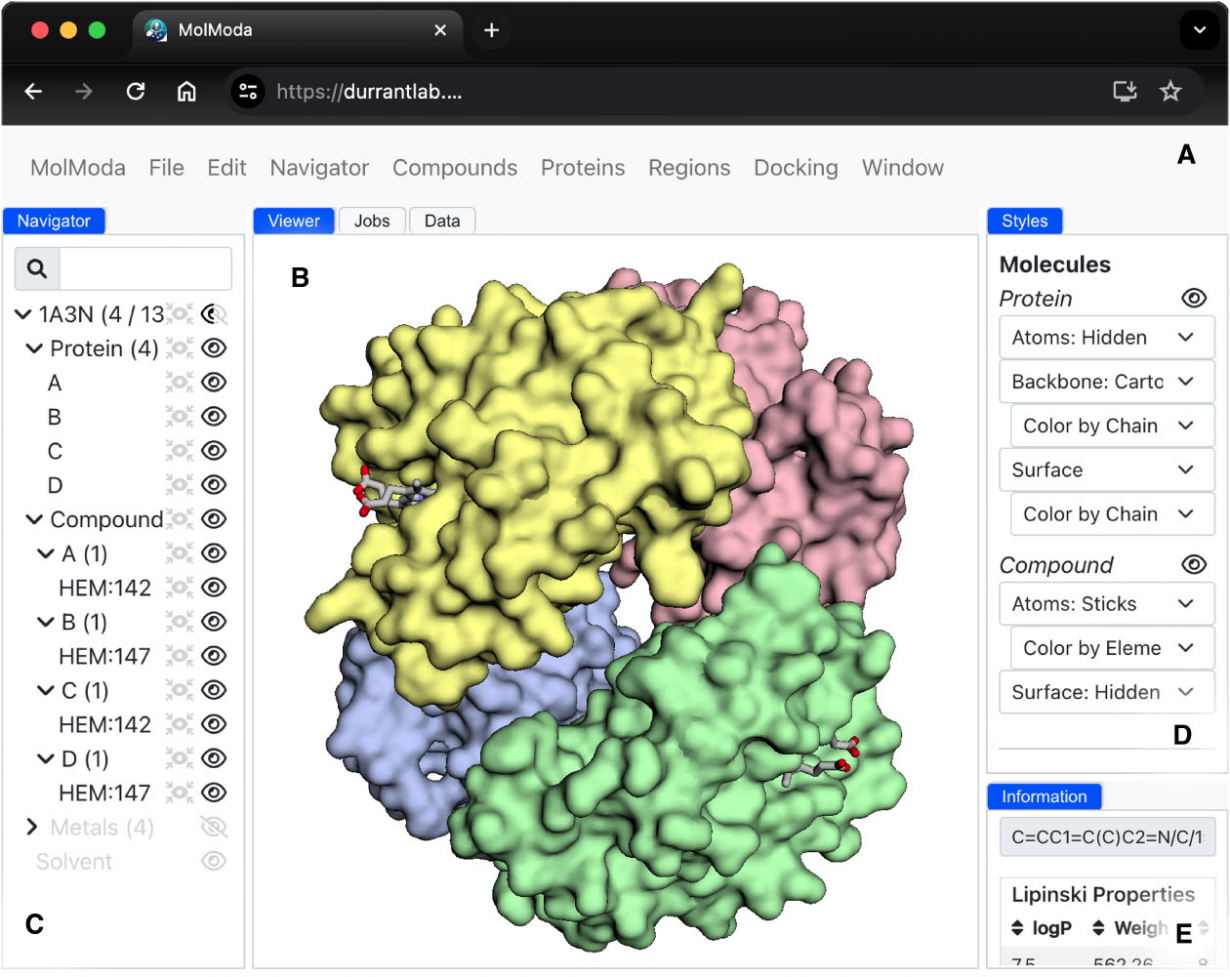
Illustration of the MolModa user interface. (**A**) The top-level menu bar provides access to the plugin tools. (**B**) The central molecular viewer displays the structures of loaded proteins and small-molecule compounds. (**C**) The hierarchical Navigator panel lists the components of each loaded molecule. (**D**) The Styles panel allows users to customize the visual representation of the molecules. (**E**) The Information panel provides information about the structure and molecular properties of the selected compound.

Users first load structures of their protein target and candidate small-molecule ligands (i.e. binding molecules). Plugins for loading molecules are accessible through the *File* menu in the top-level menu bar (Figure 1A). When the user loads a molecule, its structure appears in the central 3Dmol.js (16) molecular viewer (Figure 1B). The molecule and its components (e.g. protein chains, small-molecule ligands, etc.) also appear in the hierarchical Navigator panel (Figure 1C), and options for styling those components appear in the Styles panel (Figure 1D). If the user selects one of the small molecules, its structure and calculated molecular properties appear in the Information panel (Figure 1E).

MolModa provides several methods for importing protein structures via the *File* menu. Users can import local files from their hard drives. They can also import experimentally resolved structures directly from the Protein Data Bank (17,18), or predicted structures from the AlphaFold Protein Structure Database (19,20).

Users can also import small-molecule structure files from their local desktops. Additionally, they can import molecules from the PubChem database (21) or draw structures using a molecular editor powered by Kekule.js (22). Molecular text (e.g. SMILES strings) can also be pasted directly into the app from the system clipboard. If the imported small molecule includes a charged counterion (salt) unlikely to contribute to protein-target binding, the user may also ‘desalt’ to retain only the physiologically relevant portion.

MolModa aims to accommodate as many file formats as possible. It can load and save to its native *.molmoda* format, which stores the molecular structures and any user-specified molecule names, visual representations, analysis data, etc. To parse structures in the PDB, MCIF, PQR, XYZ and MMTF formats, MolModa uses the 3Dmol.js library (16); and to parse structures in the CIF, MOL2, SDF, PDBQT and SMI formats, it uses the cheminformatics toolkit Open Babel 3.1.1 (23).

Like many cheminformatics and CADD tools, Open Babel is written in C and C++, not JavaScript. It is easily compiled to run on desktops or remote servers, but not in web browsers. To overcome this challenge, we used the open-source Emscripten compiler toolchain (24–26) to compile the Open Babel codebase to WebAssembly (Wasm), just as one might compile computer code to run on operating systems such as Windows, MacOS or Linux (27) (see Supplementary Data for details). Wasm is a recently developed technology that runs binary executables entirely in users’ web browsers, on their local machines independent of operating system. This browser-centric approach allows MolModa to use Open Babel without having to run Open Babel on a remote server, thus eliminating the need for extensive server infrastructure, complex deployment strategies, or data transfers that could jeopardize IP protections.

#### Preparing structures of small-molecule compounds

MolModa uses the same Wasm-compiled Open Babel implementation to prepare small-molecule compounds for docking. Users should first add hydrogen atoms to the imported molecules (*Compounds* → *Protonation…*) per a user-specified pH. Molecular databases often store compounds without hydrogen atoms to conserve space, but these atoms play critical roles in protein/ligand binding by influencing hydrogen bonds, electrostatics, etc. Appropriate protonation should also reflect actual physiology (28–30); very different protonation states govern protein-ligand interactions in the stomach (pH ∼2) versus the pancreas (pH ∼8). These same protonation states also impact the calculated molecular properties displayed in the Information panel (e.g. logP; Figure 1E).

If necessary, MolModa also uses the Wasm-compiled Open Babel executable to assign 3D coordinates to small-molecule atoms. Many molecular file formats do not describe 3D geometry (e.g. SMILES). However, the spatial arrangement of atoms dictates how a ligand fits into a protein's binding site, so determining this geometry is also critical (31).

As part of preparing small-molecule compounds for docking, users may also wish to edit the molecular structures of existing compounds (e.g. to add molecular fragments such as those our DeepFrag algorithm might recommend (32,33)). The Edit Molecule plugin (*Compounds → Edit…*) provides a 2D molecular editor that allows users to modify existing molecules. The revised 2D structure is converted to a 3D compound for subsequent viewing, processing, and docking.

#### Preparing protein structures

To prepare protein structures for molecular docking, MolModa uses reduce 4.13 (34) (*Proteins* → *Protonation…*), which we similarly compiled to Wasm. Reduce adds hydrogen atoms to protein structures and adjusts the orientations of histidine, glutamine, and asparagine sidechains, whose conformations are challenging to resolve through X-ray crystallography (34–36). It determines the most probable orientation of these side chains by analyzing their chemical environments (e.g. potential hydrogen bond donors or acceptors, steric constraints, etc.). If a given orientation is suboptimal, reduce ‘flips’ the sidechain to optimize hydrogen bonding networks and minimize steric hindrance. Correctly modeling sidechain orientations is essential because sidechains can substantially influence the interactions between a protein target and its small-molecule ligands.

#### Identifying binding pockets

Users next identify the location of a pocket on the protein surface where small-molecule ligands might bind (37,38). Locating the binding pocket is crucial because it allows the docking algorithm to concentrate its search on a specific region, thereby improving computational efficiency and increasing the chances of finding biologically relevant ligand interactions.

When users know a binding pocket's location, they can manually position a ‘region’ (represented by a transparent box) over the pocket to define its location and size (*Regions* → *Add Region…*). When the pocket location is unknown, users can identify candidate pockets using a Wasm-compiled version of fpocket3 (39), a popular tool for detecting and characterizing protein cavities (*Protein* → *Detect Pockets…*). We previously published this Wasm-compiled fpocket3 implementation as a stand-alone browser app called FPocketWeb (37) and have now integrated it into MolModa. Users can adjust the locations and sizes of ‘region’ boxes to encompass their binding site of interest (e.g. to define a single site covering multiple cavities, to expand the detection area to accommodate larger ligands, etc.).

#### Performing molecular docking calculations

After preparing small-molecule/protein-target structures and an appropriate docking box, users perform the docking step itself (*Docking* → *Compound Docking…*). MolModa uses a Wasm-compiled version of AutoDock Vina 1.2.3 (Vina) (40,41), a popular open-source docking program, to dock and score small-molecule compounds entirely in the web browser, without requiring any remote computer resources (see Supporting Information for compilation details).

We previously published a Wasm-compiled Vina implementation as a stand-alone browser application called Webina (42), but MolModa docking includes several enhancements over this previous version. For example, MolModa can perform a virtual screen (VS) by docking multiple compounds automatically in succession, eliminating the need to manually initiate the docking process for each small molecule. We also compiled the MolModa docking plugin from a more recent version of the Vina codebase (1.2.3), so it is up-to-date relative to older versions of Webina. Finally, MolModa docking optionally skips compounds with too many rotatable bonds (15 by default), which can otherwise substantially slow down a VS.

Two user settings warrant specific discussion because their default behavior may differ from some users’ expectations. First, by default, MolModa does not consider solvent (i.e. water molecules) and metal cations when docking small-molecule compounds. These components are removed to allow docked compounds to occupy the same space and perhaps form many of the same interactions with the protein receptor. Users can click the ‘Count metals/solvent as part of the protein’ checkbox to include solvent and metals in the docking calculation, which, in some cases, may improve accuracy (43).

Second, by default, MolModa shows only the best-scoring pose for each compound (i.e. the pose most likely to be correct). However, the molecular docking calculation predicts multiple candidate poses per compound, and other poses may be more accurate in some cases. Users can uncheck the ‘Keep only highest-scoring pose for each compound’ checkbox to retain all predicted poses.

#### Analyzing docking performance

Assessing a docking protocol's ability to distinguish between known ligands and other (inactive or uncharacterized) compounds is crucial. MolModa allows users to quantify VS performance by calculating receiver operating characteristic (ROC) and enrichment-factor (EF) curves (*Docking → Evaluate Performance…*).

To calculate a ROC curve, MolModa first ranks the molecules by their docking scores, from the best to the worst. Then, for each possible threshold value, it calculates the true and false positive rates by considering molecules above and below the threshold to be predicted positives and negatives, respectively. Plotting the true positive rate against the false positive rate for all thresholds gives the ROC curve. The area under this curve (AUROC) is a useful metric for evaluating the overall performance of a virtual screen. Its value corresponds to the likelihood that a known-active compound will rank better than an inactive/uncharacterized compound, given any two compounds selected randomly from those included in the screen. An AUROC of 1.0 thus indicates perfect discrimination between active and other compounds, and an AUROC of 0.5 suggests random performance.

An enrichment factor focuses on the compounds with the best docking scores. It compares the fraction of actives in the set of top-scoring compounds to the overall fraction of actives in the virtual screen. For example, if a virtual screen of 1,000 compounds included 100 active molecules (10%), but eight of the top ten compounds were actives (80%), then the enrichment factor for the top-ten cutoff would be 8 (80%/10%).

Together, AUROC and enrichment factors provide complementary insights into a docking protocol's ability to effectively identify active compounds. Using these metrics, researchers can assess the quality of their docking results and make informed decisions about prioritizing compounds for further experimental testing.

### Viewing and saving the output

MolModa provides several methods for viewing and saving the docking output. As each compound finishes docking, MolModa automatically adds the docked pose to the central molecular viewer (Figure 1B), providing immediate feedback as the docking process unfolds. The docking scores are also added to the Data Panel in real-time, where users can sort by the scores and export the data for use in other analysis programs.

Via the *File* menu, users can also export the docked compounds to common structural formats (e.g. PDB, MOL2, SDF, SMILES) for external analysis or to a PNG image for use in presentations. MolModa can also export to VRML, a 3D-modeling format compatible with software such as Blender, allowing users to create more sophisticated visual representations, virtual-reality scenes, etc. (44)

### Creating the MolModa graphical user interface

We used several web technologies to create the MolModa GUI (see *MolModa → About…* for a complete list). Standard webpages are typically built using JavaScript, a programming language that does not scale well and so is poorly suited for complex codebases. To address this challenge, we created the MolModa GUI using TypeScript, a robust superset of JavaScript that incorporates optional static typing, classes, etc., to accelerate development and create maintainable codebases (45,46). TypeScript compiles to JavaScript and so is fully compatible with standard web browsers.

We used the open-source Vue.js framework to manage interface components and ensure their reactivity (47). Complex GUIs typically include many components (e.g. buttons, icons, etc.) that must react to user interactions. For example, when a user loads a molecule into MolModa via the menu system, the molecules must appear in the molecular viewer, the Navigator panel must update with the appropriate molecular components, and the Styles panel must display the proper options (Figure 1). Vue.js dramatically simplifies the creation of reusable components that react to user-initiated actions such as these.

The GoldenLayout JavaScript library is another critical component of the MolModa user interface. GoldenLayout enables resizable and draggable panels so users can customize the workspace to suit their needs. Users can easily arrange and interact with panels containing the molecular viewer, data tables, etc. This level of personalized control is particularly important in scientific research, where information accessibility can greatly impact productivity.

Finally, to enforce uniformity in design, we used the open-source Bootstrap styling library (48). Bootstrap ensures consistent styling (e.g. color, sizing and typography) across the entire application, helping to avoid inconsistent design that would otherwise leave the user disoriented.

### Case study: virtual screen targeting the TGFβ type I receptor kinase

To perform a small benchmark virtual screen (VS) targeting the TGFβ type I receptor kinase (TGFR1), we randomly selected twenty unique known-active compounds from the DUD-E database (49). We also randomly selected 80 unique DUD-E compounds from the TGFR1 decoy set, which includes compounds that generally match the TGFR1 actives in terms of molecular weight, LogP, hydrogen bond donors and acceptors, etc. (49). We used MolModa's *Compounds → Protonation…* plugin to protonate these 100 small-molecule models at pH 7.4.

We also downloaded the corresponding DUD-E receptor structure from the Protein Data Bank (PDB 3HMM (50)). We used MolModa's *Proteins → Protonation…* plugin to (i) assign hydrogen atoms and (ii) flip appropriate sidechains to optimize hydrogen bonding networks and minimize steric hindrance.

After these structure-preparation steps, we used MolModa to dock the 100 small molecules into the DUD-E-specified docking box (9 CPU cores, exhaustiveness setting of 8). We performed this VS on a MacBook Pro laptop (16-inch, 2021) running macOS Sonoma 14.4.1, with an Apple M1 Max chip and 64 GB memory (Google Chrome 124.0.6367.29).

To compare MolModa and Vina docking directly, we used MolModa to export the receptor and compound structures to the PDBQT format and performed the same VS from the command line with AutoDock Vina 1.2.3 (40,41) (same parameters and hardware).

### Case study: using MolModa to evaluate lead optimization strategies

To demonstrate how MolModa can be used in a small lead-optimization project, we first loaded a crystal structure of La-related protein 1 (LARP1; PDB 5V4R (51)) into a late-stage beta version of the software. We used MolModa to remove water molecules. We also removed all protein chains except for chain B and its associated m^7^GTP crystallographic ligand. We then exported chain B and the m^7^GTP ligand as separate PDB files.

We loaded these files into the DeepFrag web app (32,33). We used the *Delete Atom* tool to delete all atoms of the m^7^GTP nucleobase and the *Select Atom as Growing Point* tool to highlight the remaining atom most adjacent to (and previously bound to) the now deleted fragment. We then ran DeepFrag using the default parameters (32 rotations, without reflections or stepwise rotation) to predict fragment replacements. We selected five compounds from the top DeepFrag recommendations for further analysis.

We loaded these five structures into MolModa, assigned 3D atomic coordinates, and added hydrogen atoms appropriate for pH 7.4. We similarly protonated the 5V4R:B structure and the co-crystalized m^7^GTP ligand and defined a docking box encompassing the crystallographic pocket. We docked m^7^GTP and the five DeepFrag-suggested analogs using MolModa's default docking parameters, except we increased the exhaustiveness to 20.

## Results and discussion

### Comparing MolModa to similar tools

To clarify MolModa's advantages relative to other tools, we here compare it to several existing browser-accessible molecular-docking applications that are free for academic use: CB-Dock2 (10), SwissDock (11), MTiAutoDock (12), SeamDock (13) and fastDRH (14) (Table 1). Several factors set MolModa apart. For example, unlike MolModa, all five of these tools are server applications that run users’ calculations on remote computer resources. Consequently, they must store users’ data remotely and implement wait queues during heavy use. The CB-Dock2, SwissDock and MTiAutoDock servers have explicit policies indicating how long they retain user data (30, 7 and 30 days, respectively, unless users delete their data manually). We found no such policy in the SeamDock and fastDRH documentation. Surprisingly, fastDRH appears to retain all user data indefinitely and makes all results publicly accessible via its website.

**Table 1.** MolModa compared to five other browser-accessible docking tools that are free for academic use

	MolModa	CB-Dock2	SwissDock	MTiAutoDock	SeamDock	fastDRH
Remote compute^a^		X	X	X	X	X
Wait queues^b^		X	X	X	X	X
Remote data^c^		X	X	X	X	X
Data retention^d^	None	30 days	7 days	30 days	?	> 2 years
Ligand protonation^e^	X	X				X
Commercial use^f^	Unrestricted	?	Restricted	?	?	?

^a^Whether the tool runs calculations on remote computing resources.

^b^Whether the tool must implement wait queues to manage times of heavy use.

^c^Whether the tool stores users’ data on remote servers.

^d^The time user data is stored remotely.

^e^Whether the tool automatically adds hydrogen atoms to the uploaded ligand.

^f^Whether the tool can be used commercially.

Question marks indicate that we could not find the corresponding information in the associated publications, documentation, or websites.

MolModa also allows users to add hydrogen atoms to their candidate small-molecule ligands, a critical step for effective docking. In contrast, SwissDock, MTiAutoDock and SeamDock require users to protonate their small molecules in other programs. fastDRH adds hydrogen atoms automatically, but in our tests, the protonation was inappropriate for physiological pH.

Finally, MolModa is also notable in that it is free for academic and commercial use. SwissDock explicitly requires a separate license for commercial use, and the other servers do not expressly state whether commercial use is allowed.

### Case study: virtual screen targeting the TGFβ type I receptor kinase

To further demonstrate MolModa's usefulness, we performed a small benchmark virtual screen (VS) targeting the TGFβ type I receptor kinase (TGFR1), a key component in the TGF-β signaling pathway (50). We used MolModa to dock 100 small molecules (20 actives, 80 decoys) into the TGFR1 binding pocket.

**Figure 2.**
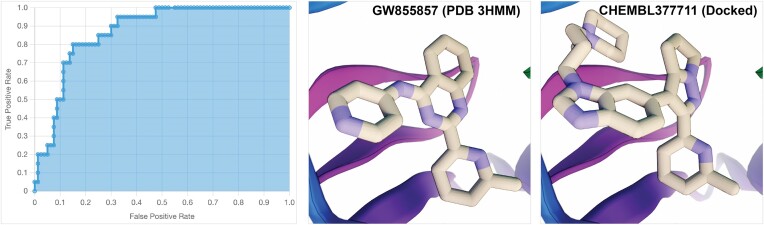
Benchmark TGFR1 virtual screen. Left panel, screenshot of the receiver operating characteristic (ROC) curve calculated using MolModa's *Docking → Evaluate Performance…* plugin. Middle panel, the crystallographic GW855857 pose (PDB 3HMM). Right panel, the MolModa-docked CHEMBL377711 pose. Both compounds place 6-methylpyridine groups and central bicyclic moieties at similar locations, lending credence to the accuracy of the docked pose. We created these images by importing MolModa-exported VRML files into the 3D modeling program Blender.

This benchmark VS was highly predictive, demonstrating how MolModa docking can effectively separate known ligands from a larger set of property-matched decoy molecules. Indeed, we specifically picked TGFR1 for testing because previous experience suggested it was well suited to Vina docking. Four of the top five ranked compounds were known actives. We used MolModa's *Docking → Evaluate Performance…* plugin to calculate the area under the receiver operating characteristic (ROC) curve (Figure 2, left panel). The value was 0.871, suggesting that if a pair of active/decoy molecules were selected at random from the set of 100 compounds, the active would have the better docking score 87.1% of the time.

To further illustrate how MolModa docking can provide valuable biological insights, we compared the poses of a co-crystallized ligand (GW855857, PDB 3HMM (50)) and the top-scoring docked compound (CHEMBL377711, a known ligand included as a positive control). To our knowledge, TGFR1 has never been co-crystalized with CHEMBL377711, but its predicted binding pose closely resembles the GW855857 crystallographic pose (Figure 2, middle and right panels). Specifically, both ligands place 6‐methylpyridine groups and central bicyclic moieties at the same positions within the binding site. MolModa affinity predictions (scores) also seem reasonable. CHEMBL377711 and GW855857 both have impressive docking scores (-12.13 and -10.78 kcal/mol, respectively), in harmony with their potent experimentally measured IC_50_ values (51 and 25 nM, respectively (52)). These results suggest that MolModa docking is effective at pose and affinity prediction for this receptor, highlighting its potential as a tool for drug discovery.

To compare MolModa and Vina docking directly, we performed the same VS from the command line with AutoDock Vina 1.2.3 (40,41). We confirmed that the MolModa and Vina docking scores were very similar (Pearson correlation coefficient of 0.93), as expected, given that the two programs were compiled from the same codebase. We attribute the slight discrepancy in scores to our use of different compilers for creating the two binaries, which can slightly affect numerical precision (42).

### Case study: using MolModa to evaluate lead optimization strategies

As a second demonstration of MolModa's usefulness, we used it in a small lead-optimization project aimed at improving the binding affinity of a known ligand via chemical substitutions. Ideally, such modifications should not disrupt existing protein/ligand interactions (i.e. the new molecule should have a similar binding pose to the original).

We focused on La-related protein 1 (LARP1), a cancer-implicated RNA-binding protein that regulates the translation of some ribosomal-protein and translation-factor mRNA transcripts (53). The 5V4R crystal structure (51) captures the LARP1 DM15 region bound to the m^7^GTP nucleotide, the first nucleotide in eukaryotic mRNA transcripts (i.e. the 5’ cap). We used MolModa to save the protein receptor and small-molecule ligand to separate files. We then loaded these files into DeepFrag, an open-source program that suggests fragment replacements and additions to improve affinity (32,33). DeepFrag generated a series of m^7^GTP analogs by replacing the m^7^GTP nucleobase with promising fragment substitutions.

**Figure 3.**
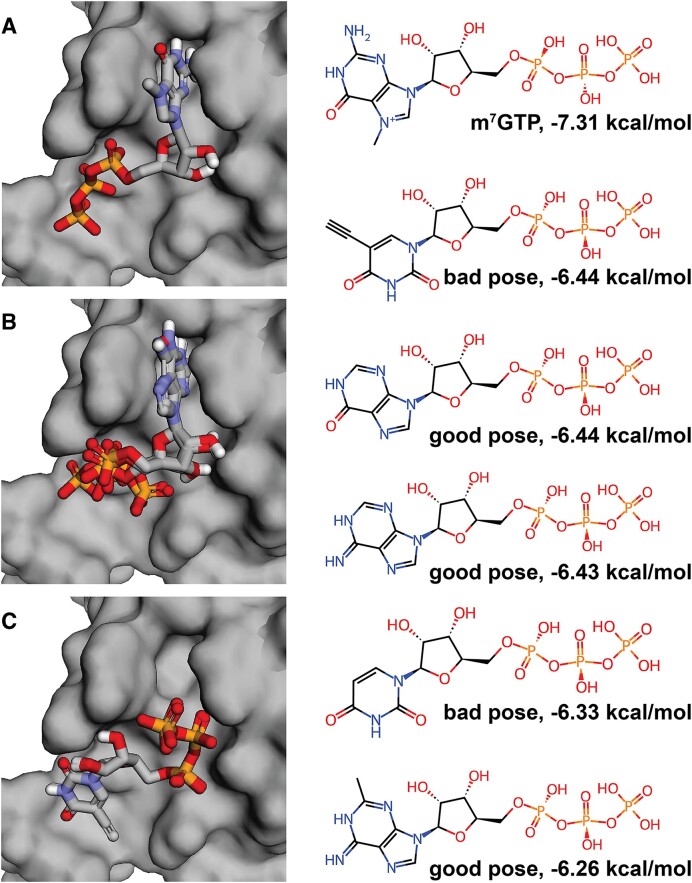
m^7^GTP and analogs docked into the LARP1 binding pocket (PDB 5V4R) using MolModa. (**A**) The docked pose of the original m^7^GTP ligand. (**B**) Three of the five DeepFrag-generated m^7^GTP analogs—all with bicyclic fragment substitutions—had m^7^GTP-similar (‘good’) docked poses. (**C**) Two additional m^7^GTP analogs—both with monocyclic fragment substitutions—had m^7^GTP-dissimilar (‘bad’) docked poses. On the right, the structures of m^7^GTP and the five analogs, an assessment of m^7^GTP-pose similarity (good/bad), and the associated docking scores.

We used MolModa to dock m^7^GTP (Figure 3A) and five of the DeepFrag-generated m^7^GTP analogs (Figures 3B and 3C) into the m^7^GTP-binding pocket. Three of the five compounds had docking poses that accurately aligned the novel fragments to the docked m^7^GTP nucleobase (Figure 3B). Interestingly, all successful substitutions had two rings, allowing them to better mimic the m^7^GTP methylated guanine (Figure 3, on the right). Though none of the analogs had a better docking score than m^7^GTP, this case study nevertheless illustrates how MolModa is helpful in evaluating different lead-optimization strategies, providing valuable insights to guide early-stage drug discovery.

### Limitations

MolModa's usability makes it an attractive tool for many projects, but it does have its limitations. Wasm-compiled programs tend to run slower than those compiled to native machine code (54). For example, in the benchmark MolModa TGFR1 VS described above, the average docking time per compound was roughly 10.1 seconds, but only 3.7 seconds when docking with Vina at the command line.

MolModa performs additional steps to improve usability, further contributing to its longer run times. Before docking a compound, MolModa automatically converts the structures to the appropriate formats, abstracting away this critical step that would otherwise complicate the user experience. After docking, MolModa automatically adds each docked molecule to its central molecular viewer, greatly simplifying visualization and analysis.

MolModa—like any docking program that runs locally—is also poorly suited to large-scale VS involving tens of thousands of compounds (or more). Such screens invariably require a computer cluster or supercomputer. Memory limitations may also impact the maximum size of a MolModa VS because the program has access only to a virtual (in-memory) file system and so cannot free up memory by saving docked poses to the disk as the VS progresses.

These limitations aside, MolModa's compelling features make it ideal for small-to-medium-sized virtual screens, especially when ease of use, quick iterative testing, and easily accessed molecular visualization justify the trade-off between speed and accessibility.

## Conclusions and outlook

In summary, MolModa implements a molecular docking workflow that runs entirely in the user's web browser. Our browser-centric approach eliminates the need for extensive remote computing resources and addresses critical data security and IP protection concerns. MolModa is as easy to access as visiting a web page, thus helping democratize access to sophisticated computational tools for drug discovery.

Future directions will include expanding the number of tools available through the MolModa interface. Specifically, we plan to introduce new features for lead optimization, cheminformatics, and molecular visualization. We are also eager to repurpose MolModa as a tool for education and outreach. Though we designed it primarily as a research tool, our focus on usability and accessibility makes it well-suited to the classroom. We plan to build a curriculum around the MolModa platform with the goal of better training the next generation of computational structural biologists and biochemists.

## Supplementary Material

gkae406_Supplemental_Files

## Data Availability

The MolModa web app is free and open to all users without requiring login. Users can access MolModa at https://durrantlab.com/molmoda. The source code is available at https://github.com/durrantlab/molmoda and https://doi.org/10.5281/zenodo.11094900 and is released under the open-source GNU General Public License v2.0.
