# Supplementary material for: MolModa: accessible and secure molecular docking in a web browser
Source: Nucleic Acids Res. 2024 May 23;52(W1):W498–506. doi: 10.1093/nar/gkae406 (PMC11223821; doi:10.1093/nar/gkae406)
Supplement: gkae406_Supplemental_Files [file gkae406_supplemental_files.zip › biotite_SI.pdf]

Compiling C/C++ Code to WebAssembly

Readers interested in how we compiled C/C++ programs to Wasm may wish to consult the git repository (https://github.com/durrantlab/molmoda/tree/main/wasm-compile), which includes examples that run in Docker containers. Here, we provide a brief, high-level explanation in hopes of helping others with their Wasm projects.

Most C/C++ projects rely on third-party libraries (dependencies). For example, these libraries may enable file input/output, parallelization to improve performance, or support for various file formats. Each dependency may have other dependencies that must be similarly resolved, further complicating the compilation process.

Two types of linking are possible when compiling a C/C++ project. Shared linking assumes the required dependencies already exist on the end user's computer, so it does not include these libraries in the binary executable. In contrast, static linking includes all required libraries, leading to larger file sizes but arguably improving the end user's experience.

The Emscripten toolchain can mimic shared linking, but such linking can impact performance in the browser and is complicated to set up. Consequently, we used static linking to compile all our projects. To reduce file sizes where possible, we excluded any libraries that were not essential (e.g., when compiling Open Babel, we did not include libraries for loading molecules in uncommon formats).

In some cases, we had to make Emscripten-specific changes to structure files (e.g., we modified some CMakeLists.txt files to switch to static linking and exclude non-critical libraries). Such modifications are common when porting projects to Wasm, a process that often involves adapting to the limitations of the browser environment.

We used Emscripten's (1) emconfigure, emcmake, and emmake for configuration, (2) emcc and emc++ for compilation, and (3) emar as the linker/archiver.

Notes on Compiling AutoDock Vina to WebAssembly

We had to make some minimal adjustments to the Vina codebase to improve MolModa compatibility. For example, to avoid having to create a separate instance of Wasm-compiled Vina in the browser for every docking run, which is prone to memory leaks, we created a wrapper function around Vina's original main function so it can dock multiple ligands with each execution. Further, to allow Wasm-compiled Vina to continue to dock subsequent ligands even when one docking run encounters an error, we replaced all the source code's `exit(EXIT_FAILURE)` calls with `throw std::runtime_error("Error message")`, which we catch in the wrapper function. These changes are detailed in the GitHub repository, which includes the Docker files we used to compile Vina: https://github.com/durrantlab/molmoda/tree/main/wasm-compile/vina. That said, no changes were made to the Vina algorithm itself.
