# Supplementary material for: MolModa: accessible and secure molecular docking in a web browser
Source: Nucleic Acids Res. 2024 May 23;52(W1):W498–506. doi: 10.1093/nar/gkae406 (PMC11223821; doi:10.1093/nar/gkae406)
Supplement: gkae406_Supplemental_Files [file gkae406_supplemental_files.zip › molmoda_2024-04-13_17-40-53.zip!/molmoda_2024-04-13_17-40-53/js/rdkitjs/GettingStartedInJS.html]

# Getting started with RDKit.js

## Drawing molecules

First we'll work with an SVG drawing:

var smiles = "CC(=O)Oc1ccccc1C(=O)O";
var mol = RDKitModule.get\_mol(smiles);
var dest = document.getElementById("example-1-output");
var svg = mol.get\_svg();
dest.outerHTML = "<div id='drawing'>" + svg + "</div>";

As of v2020.09 of the RDKit we can do the same thing using the HTML5 canvas:

var smiles = "CC(=O)Oc1ccccc1C(=O)O";
var mol = RDKitModule.get\_mol(smiles);
var canvas = document.getElementById("canvas-2");
mol.draw\_to\_canvas(canvas, -1, -1);

We can do substructure searches and highlight the results:

var smiles = "CC(=O)Oc1ccccc1C(=O)O";
var mol = RDKitModule.get\_mol(smiles);
var smarts = "Oc1[c,n]cccc1";
var qmol = RDKitModule.get\_qmol(smarts)
var mdetails = mol.get\_substruct\_match(qmol)
var canvas = document.getElementById("canvas-3");
mol.draw\_to\_canvas\_with\_highlights(canvas, mdetails);

You can also change drawing options and do highlighting with the SVG renderer,
but we don't show it here. You just need to replace

```
mol.draw_to_canvas_with_highlights(canvas, mdetails);
```

with

```
var svg = mol.get_svg_with_highlights(mdetails);
```

The same call can be used to control drawing options or to manually
set the atoms/bonds which should be highlighted:

var smiles = "CC(=O)Oc1ccccc1C(=O)O";
var mol = RDKitModule.get\_mol(smiles);
var mdetails = {};
mdetails['atoms']=[0,1,10];
mdetails['explicitMethyl'] = true;
mdetails['addAtomIndices'] = true;
mdetails['legend']='aspirin';
var canvas = document.getElementById("canvas-4");
mol.draw\_to\_canvas\_with\_highlights(canvas, JSON.stringify(mdetails));

and of course we can combine the two:

var smiles = "CC(=O)Oc1ccccc1C(=O)O";
var mol = RDKitModule.get\_mol(smiles);
var smarts = "O=C";
var qmol = RDKitModule.get\_qmol(smarts)
var mdetails = JSON.parse(mol.get\_substruct\_match(qmol));
mdetails['highlightColour'] = [1,0,1];
mdetails['legend']='aspirin';
var canvas = document.getElementById("canvas-5");
mol.draw\_to\_canvas\_with\_highlights(canvas, JSON.stringify(mdetails));

The currently supported options are:

1. atoms,bonds: lists to specify which atoms/bonds are highlighted
2. width,heightoffsetx,offsety: used to
   draw in a subregion of a canvas. Only supported by the HTML5 canvas
   drawer.
3. legend: the legend drawn under the molecule
4. These MolDrawOptions values: 
   atomLabelDeuteriumTritium,
   dummiesAreAttachments,
   circleAtoms,
   continuousHighlight,
   fillHighlights,
   highlightRadius,
   flagCloseContactsDist,
   includeAtomTags,
   clearBackground,
   legendFontSize,
   maxFontSize,
   minFontSize,
   annotationFontScale,
   fontFile,
   multipleBondOffset,
   padding,
   additionalAtomLabelPadding,
   bondLineWidth,
   scaleBondWidth,
   scaleHighlightBondWidth,
   highlightBondWidthMultiplier,
   prepareMolsBeforeDrawing,
   fixedScale,
   fixedBondLength,
   rotate,
   addAtomIndices,
   addBondIndices,
   addStereoAnnotation,
   atomHighlightsAreCircles,
   centreMoleculesBeforeDrawing,
   explicitMethyl,
   includeMetadata,
   includeRadicals,
   highlightColour,
   backgroundColour,
   legendColour,
   symbolColour,
   atomLabels

It's often useful to generate molecule renderings where the coordinates of a core is constrained.

var smiles = "c1cnc(C)nc1C(=O)O";
var mol = RDKitModule.get\_mol(smiles);
var template = `
Mrv2014 10192005332D
0 0 0 0 0 999 V3000
M V30 BEGIN CTAB
M V30 COUNTS 6 6 0 0 0
M V30 BEGIN ATOM
M V30 1 C -5.7917 2.5817 0 0
M V30 2 N -7.1253 1.8117 0 0
M V30 3 C -7.1253 0.2716 0 0
M V30 4 C -5.7917 -0.4984 0 0
M V30 5 C -4.458 0.2716 0 0
M V30 6 N -4.458 1.8117 0 0
M V30 END ATOM
M V30 BEGIN BOND
M V30 1 1 1 2
M V30 2 2 2 3
M V30 3 1 3 4
M V30 4 2 4 5
M V30 5 1 5 6
M V30 6 2 1 6
M V30 END BOND
M V30 END CTAB
M END
`;
var qmol = RDKitModule.get\_mol(template);
mol.generate\_aligned\_coords(qmol,true);
var tdetails = mol.get\_substruct\_match(qmol);
var canvas = document.getElementById("canvas-6");
mol.draw\_to\_canvas\_with\_highlights(canvas,tdetails);
