# Supplementary material for: MolModa: accessible and secure molecular docking in a web browser
Source: Nucleic Acids Res. 2024 May 23;52(W1):W498–506. doi: 10.1093/nar/gkae406 (PMC11223821; doi:10.1093/nar/gkae406)
Supplement: gkae406_Supplemental_Files [file gkae406_supplemental_files.zip › molmoda_2024-04-13_17-40-53.zip!/molmoda_2024-04-13_17-40-53/js/obabel-wasm/instance_test.html]

Test


- Home
- About
- Contact
- Test

# Test

Test
