# Supplementary material for: MolModa: accessible and secure molecular docking in a web browser
Source: Nucleic Acids Res. 2024 May 23;52(W1):W498–506. doi: 10.1093/nar/gkae406 (PMC11223821; doi:10.1093/nar/gkae406)
Supplement: gkae406_Supplemental_Files [file gkae406_supplemental_files.zip › molmoda_2024-04-13_17-40-53.zip!/molmoda_2024-04-13_17-40-53/docs/interface/info/index.html]

Information - MolModa Documentation


 


Skip to content

MolModa Documentation

Information


Initializing search

durrantlab/molmoda


- Home
- Interface
- Structures
- Tutorials

MolModa Documentation

durrantlab/molmoda

- Home
- Interface

  Interface
  - Main
  - Navigator
  - Styles
  - Information


    Information


    Table of contents
    - Molecular properties
- Structures
- Tutorials

Table of contents

- Molecular properties

# Information

The Information section of our application is a dedicated space designed to offer users immediate access to critical molecular property information.
This feature enriches the user's understanding of molecular structures through a concise and informative display, encompassing a range of data points, including 2D representations, SMILES strings, and computed molecular properties.

## Molecular properties

For more information, go to the molecular properties documentation.

Back to top


CC BY-SA 4.0 by Durrant Lab
