# Supplementary material for: MolModa: accessible and secure molecular docking in a web browser
Source: Nucleic Acids Res. 2024 May 23;52(W1):W498–506. doi: 10.1093/nar/gkae406 (PMC11223821; doi:10.1093/nar/gkae406)
Supplement: gkae406_Supplemental_Files [file gkae406_supplemental_files.zip › molmoda_2024-04-13_17-40-53.zip!/molmoda_2024-04-13_17-40-53/docs/interface/styles/index.html]

Styles - MolModa Documentation


 


Skip to content

MolModa Documentation

Styles


Initializing search

durrantlab/molmoda


- Home
- Interface
- Structures
- Tutorials

MolModa Documentation

durrantlab/molmoda

- Home
- Interface

  Interface
  - Main
  - Navigator
  - Styles


    Styles


    Table of contents
    - Cartoon
    - Surface
    - Atoms
  - Information
- Structures
- Tutorials

Table of contents

- Cartoon
- Surface
- Atoms

# Styles

MolModa's Styles feature allows users to customize the visual representation of molecular structures, including proteins and compounds.
This documentation provides an overview of the visual styles available for proteins, each designed to highlight different aspects of molecular structure for enhanced analysis and presentation.


## Cartoon

The Cartoon style abstractly represents protein structures, emphasizing the overall fold and secondary structural elements.

SpectrumSecondaryChainSolid

Displays the protein in a gradient of colors along its length, helpful in distinguishing different parts of the molecule.

Highlights the secondary structures (e.g., α-helices and β-sheets) using distinct colors and shapes.

Colors the protein based on its chain composition, aiding in identifying multi-chain complexes.

Presents the protein in a single, solid color for a simplified view.

## Surface

Surface styles provide a detailed view of the molecular surface, highlighting properties such as element types or charge distributions.

ElementCarbonsSecondary structureChainSolid

Colors the surface based on the type of elements, offering insights into the chemical composition.

Differentiates carbon atoms from other elements, useful for organic molecules analysis.

The surface is colored according to secondary structural elements, making it easy to identify helices, sheets, and coils.

Indicates the chain identity through color coding, which is helpful in visualizing protein complexes.

A uniform color for the entire surface, focusing on the shape and contours of the molecule.

## Atoms

Atom styles depict individual atoms and bonds, offering detailed insights into molecular architecture.

SticksLinesSpheres

Represents bonds as sticks and atoms as small spheres, ideal for detailed structural analysis.

Uses lines to represent bonds between atoms, providing a simplified view of the molecular structure.

Displays atoms as spheres, scaled according to their van der Waals radii, highlighting the molecular volume.

Back to top


CC BY-SA 4.0 by Durrant Lab
