# Supplementary material for: MolModa: accessible and secure molecular docking in a web browser
Source: Nucleic Acids Res. 2024 May 23;52(W1):W498–506. doi: 10.1093/nar/gkae406 (PMC11223821; doi:10.1093/nar/gkae406)
Supplement: gkae406_Supplemental_Files [file gkae406_supplemental_files.zip › molmoda_2024-04-13_17-40-53.zip!/molmoda_2024-04-13_17-40-53/docs/interface/navigator/index.html]

Navigator - MolModa Documentation


 


Skip to content

MolModa Documentation

Navigator


Initializing search

durrantlab/molmoda


- Home
- Interface
- Structures
- Tutorials

MolModa Documentation

durrantlab/molmoda

- Home
- Interface

  Interface
  - Main
  - Navigator


    Navigator


    Table of contents
    - Features
    - Utilization
    - Best Practices
  - Styles
  - Information
- Structures
- Tutorials

Table of contents

- Features
- Utilization
- Best Practices

# Navigator

The Navigator is an integral feature of MolModa.
It is designed to provide users with comprehensive control over the molecules and regions within their project.
It also acts as a dynamic inventory, listing all loaded or computed molecular structures, facilitating efficient management and interaction with these entities.

The Navigator displays all molecular entities in the system, including proteins, ligands, and any other structures that have been loaded or resulted from computational processes.
Its interface is organized to offer a clear view of the project's molecular components, making it straightforward for users to identify and select specific items for further action.

## Features

Hide/ShowFocusDelete

Users can easily toggle the visibility of structures or regions.
This feature allows for a clutter-free visualization space in the viewer, focusing only on elements of interest at any given time.

This function enables users to center the view on a selected structure or region within the viewer.
It's particularly useful for examining detailed interactions or configurations of molecular structures, ensuring the selected entity is prominently displayed.

When necessary, users can remove structures or regions from the project.
This action is irreversible and should be used to eliminate unneeded or erroneously loaded components, helping maintain the project's integrity and focus.

## Utilization

The Navigator's design emphasizes ease of use, allowing for intuitive navigation and manipulation of molecular structures:

- **Accessing the Navigator:** The Navigator panel is easily accessible from the main window, typically located on left side for convenient access.
- **Managing Structures:** By clicking on an entity within the Navigator, users are presented with options to hide/show, center, or delete, which can be executed with a simple click.
- **Organizing Projects:** Through the Navigator, users can effectively manage and organize their molecular structures, enhancing the overall workflow and analysis process within the application.

## Best Practices

- **Regularly Review:** Keep the Navigator organized by regularly reviewing and managing the structures and regions, ensuring only relevant entities are present.
- **Use Hide/Show:** Leverage the hide/show functionality to focus on specific areas of interest without permanently removing structures from your project.
- **Caution with Deletion:** Use the delete function judiciously, considering the permanence of this action and its impact on your project's data.

Back to top


CC BY-SA 4.0 by Durrant Lab
