# Supplementary material for: MolModa: accessible and secure molecular docking in a web browser
Source: Nucleic Acids Res. 2024 May 23;52(W1):W498–506. doi: 10.1093/nar/gkae406 (PMC11223821; doi:10.1093/nar/gkae406)
Supplement: gkae406_Supplemental_Files [file gkae406_supplemental_files.zip › molmoda_2024-04-13_17-40-53.zip!/molmoda_2024-04-13_17-40-53/docs/interface/log/index.html]

Log - MolModa Documentation


 


Skip to content

MolModa Documentation

Log


Initializing search

durrantlab/molmoda


- Home
- Interface
- Structures
- Tutorials

MolModa Documentation

durrantlab/molmoda

- Home
- Interface

  Interface
  - Main
  - Navigator
  - Styles
  - Information
- Structures
- Tutorials

# Log

TODO:

Back to top


CC BY-SA 4.0 by Durrant Lab
