# Supplementary material for: MolModa: accessible and secure molecular docking in a web browser
Source: Nucleic Acids Res. 2024 May 23;52(W1):W498–506. doi: 10.1093/nar/gkae406 (PMC11223821; doi:10.1093/nar/gkae406)
Supplement: gkae406_Supplemental_Files [file gkae406_supplemental_files.zip › molmoda_2024-04-13_17-40-53.zip!/molmoda_2024-04-13_17-40-53/docs/interface/main/index.html]

Main - MolModa Documentation


 


Skip to content

MolModa Documentation

Main


Initializing search

durrantlab/molmoda


- Home
- Interface
- Structures
- Tutorials

MolModa Documentation

durrantlab/molmoda

- Home
- Interface

  Interface
  - Main


    Main


    Table of contents
    - Viewer
    - Jobs
    - Data
  - Navigator
  - Styles
  - Information
- Structures
- Tutorials

Table of contents

- Viewer
- Jobs
- Data

# Main

The main window is the core interface of our application, designed to provide quick access to critical project components: the viewer, jobs, and data.
This design ensures users can manage their molecular research projects effectively.

## Viewer

The viewer component is essential for visualizing molecules, such as proteins and compounds.
Through the styles window, it offers a range of customizable visual representations, allowing for detailed and specific views of molecular structures.
Functionality integrated with the navigator facilitates the selective display and updating of molecule visuals within the viewer, enhancing the interactive experience.


## Jobs

Within the jobs section, users can track the progress of tasks in real-time.
This includes ongoing jobs like molecular docking, their completion status, and runtime.
A job history feature is also available, providing a record of past jobs for analysis and review.


## Data

The data section organizes critical information from calculations including details on binding pockets, compound properties, docking scores, and poses.
It features sortable and filterable tables, making it easier for users to access and analyze their data efficiently, supporting informed decision-making in their projects.

PocketDocking

Back to top


CC BY-SA 4.0 by Durrant Lab
