# Supplementary material for: MolModa: accessible and secure molecular docking in a web browser
Source: Nucleic Acids Res. 2024 May 23;52(W1):W498–506. doi: 10.1093/nar/gkae406 (PMC11223821; doi:10.1093/nar/gkae406)
Supplement: gkae406_Supplemental_Files [file gkae406_supplemental_files.zip › molmoda_2024-04-13_17-40-53.zip!/molmoda_2024-04-13_17-40-53/docs/index.html]

MolModa Documentation


 


Skip to content

MolModa Documentation

Home


Initializing search

durrantlab/molmoda


- Home
- Interface
- Structures
- Tutorials

MolModa Documentation

durrantlab/molmoda

- Home


  Home


  Table of contents
  - Key features
- Interface
- Structures
- Tutorials

Table of contents

- Key features

# MolModa

MolModa is at the forefront of innovation in early-stage drug discovery, offering a secure, user-friendly platform that democratizes molecular docking.
Designed to run entirely within your web browser, MolModa uses advanced computational techniques to predict how small molecules, potential drug candidates, bind to target proteins.
This powerful tool accelerates identifying and refining new pharmaceuticals by providing critical insights for prioritizing compounds for experimental testing.

## Key features

MolModa streamlines the molecular docking process into manageable steps, from loading and converting molecular structures to performing docking calculations and analyzing the results.
With support for various file formats and integration with key databases, MolModa ensures that your research benefits from the latest advances in cheminformatics and structural biology.

- **Accessibility:** No installations or downloads required—get started directly in your web browser.
- **Security:** Full data privacy with all computations performed locally, ensuring intellectual property protection.
- **User-Friendly:** An intuitive graphical user interface simplifies the molecular docking workflow for experts and non-experts alike.
- **Open Source:** Contributing to the scientific community with source code available on GitHub under the GNU General Public License v2.0.

Back to top


CC BY-SA 4.0 by Durrant Lab
