# Supplementary material for: MolModa: accessible and secure molecular docking in a web browser
Source: Nucleic Acids Res. 2024 May 23;52(W1):W498–506. doi: 10.1093/nar/gkae406 (PMC11223821; doi:10.1093/nar/gkae406)
Supplement: gkae406_Supplemental_Files [file gkae406_supplemental_files.zip › molmoda_2024-04-13_17-40-53.zip!/molmoda_2024-04-13_17-40-53/docs/structures/molprop/weight/index.html]

Molecular weight - MolModa Documentation


 


Skip to content

MolModa Documentation

Molecular weight


Initializing search

durrantlab/molmoda


- Home
- Interface
- Structures
- Tutorials

MolModa Documentation

durrantlab/molmoda

- Home
- Interface
- Structures


  Structures
  - Loading
  - Molecular properties

    Molecular properties
    - logP
    - Molecular weight


      Molecular weight


      Table of contents
      - Drug Absorption and Distribution
      - Solubility
      - Metabolism and excretion

        - Biotransformation
        - Renal excretion
      - Drug Efficacy and Toxicity

        - Target Binding
        - Toxicity Potential
      - Formulation and Delivery

        - Drug Formulation
        - Administration Route
    - Hydrogen bonding
    - Molar refractivity
    - SP3-hybridized carbons
    - Surface area
    - Topological polar surface area
  - Pockets
- Tutorials

Table of contents

- Drug Absorption and Distribution
- Solubility
- Metabolism and excretion

  - Biotransformation
  - Renal excretion
- Drug Efficacy and Toxicity

  - Target Binding
  - Toxicity Potential
- Formulation and Delivery

  - Drug Formulation
  - Administration Route

# Molecular weight

Molecular weight (MW) plays a crucial role in computer-aided drug design (CADD) and has implications for drug absorption, distribution, metabolism, excretion (ADME), efficacy, toxicity, formulation, and delivery.
Understanding and analyzing the MW of compounds is crucial in the early stages of drug discovery and development.

## Drug Absorption and Distribution

One of the foundational principles that highlight the importance of molecular weight in the development of orally active drugs is Lipinski's Rule of Five.
This rule posits that for a compound to be orally active and efficiently absorbed by the human body, it should ideally have a molecular weight of less than 500 grams per mole (g/mol).
The rationale behind this guideline is that compounds with higher molecular weights may encounter difficulties in absorption and permeation through biological membranes.
This insight serves as a crucial checkpoint in the early stages of drug development, steering researchers towards compounds with a higher likelihood of successful oral administration.

Furthermore, the concept of size and cell permeability elaborates on the relationship between a compound's molecular weight and its ability to traverse cell membranes.
Molecular size, which is intrinsically linked to molecular weight, plays a pivotal role in determining a molecule's absorption efficiency.
Smaller molecules, due to their lower molecular weight, typically exhibit better absorption characteristics because they can more easily penetrate the lipid bilayer of cell membranes.
This penetration is essential for the drug to reach its target sites within the body effectively.
In contrast, larger molecules might struggle to cross these barriers, limiting their therapeutic utility when oral administration is desired.

## Solubility

Solubility prediction stands as a significant application of molecular weight (MW) analysis, wherein the MW of a compound is closely scrutinized to infer its solubility characteristics.
Compounds with lower molecular weights are generally found to be more soluble in water.
This correlation between MW and solubility is pivotal, as water solubility is directly linked to a drug's ability to be absorbed into the bloodstream upon oral ingestion.

The principle that underlies this relationship is grounded in the physical chemistry of molecules.
Water solubility is influenced by a compound's ability to form hydrogen bonds with water molecules, a process more efficiently achieved by smaller molecules.
As the molecular weight of a compound decreases, it typically presents less steric hindrance, allowing for closer interaction with water molecules.
Consequently, these lower MW compounds can dissolve more readily, enhancing their availability to be absorbed from the gastrointestinal tract into the systemic circulation.

This insight into solubility prediction based on molecular weight is a cornerstone of drug design and optimization.
It guides pharmaceutical chemists and drug developers in modifying the chemical structure of lead compounds to reduce their molecular weight, thereby increasing their solubility and, by extension, their oral bioavailability.
The ability to predict and enhance solubility through MW analysis is especially critical in the early stages of drug development, where the selection of compounds with favorable pharmacokinetic properties can significantly impact the success of subsequent clinical phases.

## Metabolism and excretion

This understanding is deeply influenced by the molecular weight (MW) of the compound, which plays a significant role in both its biotransformation and renal excretion.
These factors are essential in assessing a drug's pharmacokinetic profile, which in turn influences dosage, administration frequency, and potential for toxicity.

### Biotransformation

Biotransformation refers to the metabolic processes that drugs undergo within the body, primarily in the liver, to become more water-soluble and thus easier to excrete.
Compounds with higher molecular weights are generally more susceptible to enzymatic metabolism because they offer more chemical groups that can be modified by metabolic enzymes.
This susceptibility allows such compounds to be transformed into metabolites that are more readily excreted.
However, an interesting paradox arises with very large molecules.
Despite being more amenable to enzymatic action due to their size, they may actually be poorly metabolized.
This poor metabolism is due to the molecule's size and complexity, which can hinder the enzyme's ability to access specific sites on the molecule for metabolism, potentially leading to accumulation in the body and adverse effects.

### Renal excretion

Renal excretion, the process by which drugs and their metabolites are eliminated from the body through the kidneys, is also significantly influenced by a compound's molecular weight.
Generally, molecules with a MW below 300-500 g/mol are more readily excreted by the kidneys.
This is because smaller molecules can more easily pass through the glomerular filtration barrier, a key step in the renal excretion process.
Compounds exceeding this MW range are less likely to be excreted unchanged and may require metabolic transformation into smaller, more water-soluble metabolites for elimination.
The threshold MW range is crucial for drug developers to consider, as it influences not just the drug's efficacy but also its potential for causing toxicity due to accumulation.

## Drug Efficacy and Toxicity

The drug's therapeutic potential, can be significantly influenced by the drug's molecular weight (MW).
Moreover, the MW also bears implications for the drug's safety profile, particularly concerning its toxicity and potential to elicit immune responses.
Understanding these aspects is critical for the development of effective and safe therapeutic agents.

### Target Binding

The process of target binding is fundamental to a drug's action, where the molecular structure of the drug interacts with a specific biological target, such as a protein, to induce a therapeutic effect.
The molecular weight of a compound can greatly influence this interaction. Larger molecules, owing to their size, have more potential interaction points with the target protein.
This increased number of interaction points can enhance the binding affinity and specificity of the drug to its target, which is often desirable for therapeutic efficacy.
However, optimizing this aspect requires a delicate balance; while additional interaction points can improve efficacy, they may also complicate the drug design process by increasing the complexity of achieving selective binding.
The challenge lies in designing large molecules that can not only bind effectively to their intended target but also maintain a high degree of specificity to minimize off-target effects.

### Toxicity Potential

The safety profile of a drug is as crucial as its efficacy, with the potential for toxicity being a primary concern. Larger molecules carry a higher risk of toxicity and immune responses, partially due to their complex structures.
The complexity of larger drugs increases the likelihood of off-target interactions, where the drug may inadvertently bind to unintended proteins or receptors, potentially leading to adverse effects.
Additionally, the immune system may recognize these large, complex molecules as foreign entities, triggering immune responses that can range from mild to severe.
This recognition can complicate the development of large-molecule drugs, necessitating thorough preclinical and clinical evaluations to assess and mitigate risks of toxicity and immunogenicity.

## Formulation and Delivery

The molecular weight (MW) of a compound plays a critical role in both drug formulation and the determination of the most appropriate route of administration.
These aspects are vital for ensuring that the drug can be delivered to its site of action in the most effective manner, thus optimizing its therapeutic potential while minimizing side effects.

### Drug Formulation

The formulation of a drug involves creating a drug product that is safe, effective, and can be consistently manufactured.
For compounds with high molecular weights, the challenge often lies in enhancing their bioavailability—the proportion of the drug that enters the circulation when introduced into the body and is able to have an active effect.
High MW compounds typically have poor solubility in water, which can limit their absorption from the gastrointestinal tract if administered orally. As a result, such compounds may require specialized delivery systems to improve their bioavailability.
These systems can include nanoparticle-based carriers, liposomes, or other advanced drug delivery technologies that enhance the solubility or facilitate the targeted delivery of the drug to its intended site of action.
The development of these specialized delivery systems is a complex and crucial aspect of pharmaceutical sciences, requiring a deep understanding of material science, chemistry, and biology to create formulations that overcome the limitations imposed by the compound's molecular weight.

### Administration Route

The choice of administration route for a drug is significantly influenced by its molecular weight, among other factors.
Different administration routes can affect the drug's absorption, distribution, metabolism, and excretion, ultimately impacting its efficacy and safety.
For instance, orally administered drugs — the most convenient and commonly preferred route — are ideal for compounds with relatively lower molecular weights that are readily absorbed through the gastrointestinal tract.

In contrast, high MW compounds, which may have poor oral bioavailability due to limited solubility or permeability, might be better suited for alternative routes of administration, such as intravenous, subcutaneous, or intramuscular injections.
These alternative routes can bypass the barriers to absorption encountered in the gastrointestinal tract, providing a more direct path to the bloodstream and the site of action.
The decision on the administration route takes into consideration the drug's physicochemical properties, intended action, and the target patient population's needs, aiming to maximize the therapeutic benefits while ensuring patient convenience and compliance.

Back to top


CC BY-SA 4.0 by Durrant Lab
