# Supplementary material for: MolModa: accessible and secure molecular docking in a web browser
Source: Nucleic Acids Res. 2024 May 23;52(W1):W498–506. doi: 10.1093/nar/gkae406 (PMC11223821; doi:10.1093/nar/gkae406)
Supplement: gkae406_Supplemental_Files [file gkae406_supplemental_files.zip › molmoda_2024-04-13_17-40-53.zip!/molmoda_2024-04-13_17-40-53/docs/structures/molprop/logp/index.html]

logP - MolModa Documentation


 


Skip to content

MolModa Documentation

logP


Initializing search

durrantlab/molmoda


- Home
- Interface
- Structures
- Tutorials

MolModa Documentation

durrantlab/molmoda

- Home
- Interface
- Structures


  Structures
  - Loading
  - Molecular properties

    Molecular properties
    - logP


      logP


      Table of contents
      - Interpretation

        - Drug Absorption and permeability
        - Solubility and Formulation
        - Toxicity and Side Effects
      - Method

        - Atom classification
        - Calculation
    - Molecular weight
    - Hydrogen bonding
    - Molar refractivity
    - SP3-hybridized carbons
    - Surface area
    - Topological polar surface area
  - Pockets
- Tutorials

Table of contents

- Interpretation

  - Drug Absorption and permeability
  - Solubility and Formulation
  - Toxicity and Side Effects
- Method

  - Atom classification
  - Calculation

# logP

The logP value, representing a compound's partition coefficient between octanol and water, is a critical parameter in computer-aided drug design (CADD) and various molecular applications due to its direct correlation with a compound`s lipophilicity.

Note

Lipophilicity is a key factor influencing a molecule`s absorption, distribution, metabolism, excretion, and toxicity (ADMET) properties, which are essential in drug discovery.

## Interpretation

Here`s how the significance of the logP value can be interpreted in these contexts.

### Drug Absorption and permeability

In the context of drug absorption and permeability, the lipophilicity of a compound, as indicated by its logP value, plays a pivotal role.
A compound must strike a balance in lipophilicity to effectively permeate cell membranes and achieve optimal oral bioavailability; typically, a logP value between 2 and 5 is associated with favorable absorption characteristics.

This balance is crucial for general drug absorption and for penetration of the blood-brain barrier (BBB).
Specifically, drugs designed to act on the central nervous system (CNS) require a higher degree of lipophilicity to cross the BBB and reach their target site within the brain.
Thus, a suitable logP value is indispensable in developing CNS drugs, ensuring they possess the necessary characteristics to cross the BBB effectively and exhibit the desired therapeutic action once they reach the central nervous system.

### Solubility and Formulation

The relationship between a compound's logP value and its aqueous solubility is inversely proportional, with high logP values often signaling poor water solubility.
This presents significant challenges in the formulation and delivery of drugs, as a balance must be struck between lipophilicity for effective cellular absorption and aqueous solubility for systemic availability.
Understanding and optimizing the logP value is thus critical in developing drug formulations that achieve this balance.

Moreover, the logP value can inform the choice of formulation strategies.
Specifically, it can guide the selection of appropriate excipients and delivery systems that enhance the solubility of lipophilic drugs.
This, in turn, improves the bioavailability of these drugs, ensuring that they can be effectively absorbed into the bloodstream and reach their intended targets within the body.

### Toxicity and Side Effects

In drug development, accurately predicting and managing the toxicity and side effects of potential pharmaceutical compounds is of paramount importance.
Compounds characterized by very high logP values pose a particular concern, as they may preferentially accumulate in lipid-rich tissues, potentially leading to adverse toxicity levels.
This underscores the importance of closely monitoring and optimizing the logP values throughout the drug design process to mitigate such risks effectively.

Furthermore, a nuanced understanding of how a compound's logP value influences its interactions with biological targets enables scientists to modify the drug's chemical structure judiciously.
Such strategic modifications aim to minimize unwanted interactions that could result in side effects, thereby enhancing the drug`s therapeutic index.
This dual focus on minimizing toxicity while maximizing therapeutic efficacy exemplifies the critical role of logP in developing safer, more effective drugs.

## Method

MolModa computes the octanol−water partition coefficient (logP) for molecules using the method described by Wildman and Crippen ^1^.

### Atom classification

The approach relies on classifying atoms into one of 68 predefined atom types, each associated with a specific contribution to the molecule's logP value.
Atoms are classified based on their immediate chemical environment, considering the neighboring atoms' type, aromaticity, and bonding.
This classification reduces ambiguity and ensures a consistent assignment of atom types across different molecules.

Atom Type Descriptions and Contributions

| type | descriptions | SMARTS | logP \(\alpha\_i\) |
| --- | --- | --- | --- |
| C1 | 1°, 2° aliphatic | `[CH4]`, `[CH3]C`, `[CH2](C)C` | 0.1441 |
| C2 | 3°, 4° aliphatic | `[CH](C)(C)C`, `[C](C)(C)(C)C` | 0.0000 |
| C3 | 1°, 2° | `[CH3][(N,O,P,S,F,Cl,Br,I)]`, `[CH3][A#N]`, `[CH2X4][A#N]`, `[CH3][#15]`, `[CH2X4][#15]`, `[CH3][#16]`, `[CH2X4][#16]`, `[CH3][#53]`, `[CH2X4][#53]`, `!([CH2X4]a)` | -0.2035 |
|  | heteroatom | `[CH2X4][(N,O,P,S,F,Cl,Br,I)]` |  |
| C4 | 3°, 4° | `[CH1X4][(N,O,P,S,F,Cl,Br,I)]`, `[CHX4][A#N]`, `[CH0X4][A#N]`, `[CHX4][#15]`, `[CH0X4][#15]`, `[CHX4][#16]`, `[CH0X4][#16]`, `[CHX4][#53]`, `!([CH0X4][#53])`, `!([CHX4]a)` or `!([CH0X4]a)` | -0.2051 |
|  | heteroatom | `[CH0X4][(N,O,P,S,F,Cl,Br,I)]` |  |
| C5 | C = heteroatom | `[C] = [A#X]` | -0.2783 |
| C6 | C = C aliphatic | `[CH2] = C`, `[CH1](=C)A`, `[CH0](=C)(A)A`,`[C](=C)=C` | 0.1551 |
| C7 | acetylene, nitrile | `[CX2]#A` | 0.00170 |
| C8 | 1° aromatic carbon | `[CH3]c` | 0.08452 |
| C9 | 1° aromatic heteroatom | `[CH3][a#X]` | -0.1444 |
| C10 | 2° aromatic | `[CH2X4]a` | -0.0516 |
| C11 | 3° aromatic | `[CHX4]a` | 0.1193 |
| C12 | 4° aromatic | `[CH0X4]a` | -0.0967 |
| C13 | aromatic heteroatom | `[cH0]−[!(C,N,O,S,F,Cl,Br,I)]`, `[c][#5]`, `[c][#14]`, `[c][#15]`, `[c][#33]`, `[c][#34]`, `[c][#50]`, `[c][#80]` | -0.5443 |
| C14 | aromatic halide | `[c][#9]` | 0.0000 |
| C15 | aromatic halide | `[c][#17]` | 0.2450 |
| C16 | aromatic halide | `[c][#35]` | 0.1980 |
| C17 | aromatic halide | `[c][#53]` | 0.0000 |
| C18 | aromatic | `[cH]` | 0.1581 |
| C19 | aromatic bridgehead | `[c](:a)(:a):a` | 0.2955 |
| C20 | 4° aromatic | `[c](:a)(:a)-a` | 0.2713 |
| C21 | 4° aromatic | `[c](:a)(:a)-C` | 0.1360 |
| C22 | 4° aromatic | `[c](:a)(:a)-N` | 0.4619 |
| C23 | 4° aromatic | `[c](:a)(:a)-O` | 0.5437 |
| C24 | 4° aromatic | `[c](:a)(:a)-S` | 0.1893 |
| C25 | 4° aromatic | `[c](:a)(:a) = C`, `[c](:a)(:a) = N`, `[c](:a)(:a) = O` | -0.8186 |
| C26 | C = C aromatic | `[C](=C)(a)A`, `[C](=C)(c)a`, `[CH](= C)a`, `[C] = c` | 0.2640 |
| C27 | aliphatic heteroatom | `[CX4][!(C,N,O,P,S,F,Cl,Br,I)]`, `[CX4][#X]`, `!([CX4][#N])`, `[CX4][#16]`, `[CX4][#15]`, `[CX4][#53]` | 0.2148 |
| CS | carbon supplemental | `[#6]` not matching any basic C type | 0.08129 |
| H1 | hydrocarbon | `[#1][#6]`, `[#1][#1]` | 0.1230 |
| H2 | alcohol | `[#1]O[CX4]`, `[#1]Oc`, `[#1]O[!(C,N,O,S)]`, `[#1][!(C,N,O)]`, `[#1]O[CX4]`, `[#1]Oc`, `[#1]O[#1]`, `[#1]O[#5]`, `[#1]O[#14]`, `[#1]O[#15]`, `[#1]O[#33]`, `[#1]O[#50]`, `[#1][#5]`, `[#1][#14]`, `[#1][#15]`, `[#1][#16]`, `[#1][#50]` | -0.2677 |
| H3 | amine | `[#1][#7]`, `[#1]O[#7]` | 0.2142 |
| H4 | acid | `[#1]OC = [#6]`, `[#1]OC = [#7]`, `[#1]OC = O`, `[#1]OC = S`, `[#1]OO`, `[#1]OS` | 0.2980 |
| HS | hydrogen supplemental | `[#1]` not matching any basic H type | 0.1125 |
| N1 | 1° amine | `[NH2+0]A` | -1.0190 |
| N2 | 2° amine | `[NH+0](A)A` | -0.7096 |
| N3 | 1° aromatic amine | `[NH2+0]a` | -1.0270 |
| N4 | 2° aromatic amine | `[NH+0](A)a`, `[NH+0](a)a` | -0.5188 |
| N5 | imine | `[NH+0] = A`, `[NH+0] = a` | 0.08387 |
| N6 | substituted imine | `[N+0](=A)A`, `[N+0](=A)a`, `[N+0](=a)A`, `[N+0](=a)a` | 0.1836 |
| N7 | 3° amine | `[N+0](A)(A)A` | -0.3187 |
| N8 | 3° aromatic amine | `[N+0](a)(A)A`, `[N+0](a)(a)A`, `[N+0](a)(a)a` | -0.4458 |
| N9 | nitrile | `[N+0]#A` | 0.01508 |
| N10 | protonated amine | `[NH3+*]`, `[NH2+*]`, `[NH+*]` | -1.950 |
| N11 | unprotonated aromatic | `[n+0]` | -0.3239 |
| N12 | protonated aromatic | `[n+*]` | -1.119 |
| N13 | 4° amine | `[NH0+*](A)(A)(A)A`, `[NH0+*](=A)(A)A`, `[NH0+*](=A)(A)a`, `[NH0+*](=[#6])=[#7]` | -0.3396 |
| N14 | other ionized nitrogen | `[N+*]#A`, `[N−*]`, `[N+*](=[N−*])=N` | 0.2887 |
| NS | nitrogen supplemental | `[#7]` not matching any basic N type | -0.4806 |
| O1 | aromatic | `[o]` | 0.1552 |
| O2 | alcohol | `[OH]`, `[OH2]` | -0.2893 |
| O3 | aliphatic ether | `[O](C)C`, `[O](C)[A#X]`, `[O]([A#X])[A#X]` | -0.0684 |
| O4 | aromatic ether | `[O](A)a`,`[O](a)a` | -0.4195 |
| O5 | oxide | `[O]=[#8]`, `[O]=[#7]`, `[OX1−*][#7]` | 0.0335 |
| O6 | oxide | `[OX1−*][#16]` | -0.3339 |
| O7 | oxide | `[OX1−*][!(N,S)]`, `[OX1−*][#15]`, `[OX1−*][#33]`, `[OX1−*][#43]`, `[OX1−*][#53]` | -1.189 |
| O8 | aromatic carbonyl | `[O]=c` | 0.1788 |
| O9 | carbonyl aliphatic | `[O]=[CH]C`, `[O]=C(C)C`, `[O]=C(C)[A#X]`,`[O]=[CH]N`, `[O]=[CH]O`,`[O]=[CH2]`, `[O]=[CX2]=O` | -0.1526 |
| O10 | carbonyl aromatic | `[O]=[CH]c`, `[O]=C(C)c`, `[O]=C(c)c`, `[O]=C(c)[a#X]`, `[O]=C(c)[A#X]`, `[O]=C(C)[a#X]` | 0.1129 |
| O11 | carbonyl heteroatom | `[O]=C([A#X])[A#X]`, `[O]=C([A#X])[a#X]`, `[O]=C([a#X])[a#X]` | 0.4833 |
| O12 | acid | `[O−1]C(=O)` | -1.326 |
| OS | oxygen supplemental | `[#8]` not matching any basic O type | -0.1188 |
| F | fluorine | `[#9−0]` | 0.4202 |
| Cl | chlorine | `[#17−0]` | 0.6895 |
| Br | bromine | `[#35−0]` | 0.8456 |
| I | iodine | `[#53−0]` | 0.8857 |
| Hal | ionic halogens | `[#9−*]`, `[#17−*]`, `[#35−*]`, `[#53−*]`, `[#53+*]` | -2.996 |
| P | phosphorous | `[#15]` | 0.8612 |
| S1 | aliphatic | `[S−0]` | 0.6482 |
| S2 | ionic sulfur | `[S−*]`, `[S+*]` | -0.0024 |
| S3 | aromatic | `[s]` | 0.6237 |
| Me1 | `B`, `Si`, `Ga`, `Ge`, `As`, `Se`, `Sn`, `Te`, `Pb`, `Ne`, `Ar`, `Kr`, `Xe`, `Rn` |  | -0.3808 |
| Me2 | `Fe`, `Cu`, `Zn`, `Tc`, `Cd`, `Pt`, A`u,`Hg` |  | -0.0025 |

These SMARTS strings are based on v1988.10 Molecular Operating Environment (MOE).

Reproduced from Wildman and Crippen ^1^.

### Calculation

The logP of a molecule is calculated as the sum of the contributions of its constituent atoms, allowing for the estimation of its lipophilicity.
The logP value of a molecule is calculated using

\[ \log \, P = \sum\_{i} n\_{i} \alpha\_{i} \]

where

- \(n\_{i}\) is the number of atoms of type \(i\) present in the molecule, and
- \(\alpha\_{i}\) is the contribution to logP for atom type \(i\).

Warning

Please be aware that the logP value of a molecule is dependent on its protonation state.
When you predict the logP value for a molecule and subsequently change its protonation state, the logP value will also change accordingly.
This is evident in the Atom Classification table above, where there are specific contributions for hydrogens.

It is essential to consider the protonation state of your molecule when interpreting and comparing logP values. The same molecule can have different logP values depending on its protonation state under specific pH conditions.
Remember this when using predicted logP values to make decisions or comparisons, especially if you have modified your molecule's protonation state.

---

1. Wildman, S. A., & Crippen, G. M. (1999). Prediction of physicochemical parameters by atomic contributions. *Journal of chemical information and computer sciences, 39*(5), 868-873. DOI: 10.1021/ci990307l ↩↩

Back to top


CC BY-SA 4.0 by Durrant Lab
