# Supplementary material for: MolModa: accessible and secure molecular docking in a web browser
Source: Nucleic Acids Res. 2024 May 23;52(W1):W498–506. doi: 10.1093/nar/gkae406 (PMC11223821; doi:10.1093/nar/gkae406)
Supplement: gkae406_Supplemental_Files [file gkae406_supplemental_files.zip › molmoda_2024-04-13_17-40-53.zip!/molmoda_2024-04-13_17-40-53/docs/structures/molprop/hb/index.html]

Hydrogen bonding - MolModa Documentation


 


Skip to content

MolModa Documentation

Hydrogen bonding


Initializing search

durrantlab/molmoda


- Home
- Interface
- Structures
- Tutorials

MolModa Documentation

durrantlab/molmoda

- Home
- Interface
- Structures


  Structures
  - Loading
  - Molecular properties

    Molecular properties
    - logP
    - Molecular weight
    - Hydrogen bonding


      Hydrogen bonding


      Table of contents
      - Definition
      - Role and interpretation
    - Molar refractivity
    - SP3-hybridized carbons
    - Surface area
    - Topological polar surface area
  - Pockets
- Tutorials

Table of contents

- Definition
- Role and interpretation

# Hydrogen bonding

Understanding the delicate dance between hydrogen bond acceptors and donors is fundamental for predicting how compounds behave within biological systems.
These interactions, pivotal for their influence on molecular characteristics such as solubility and permeability, guide therapeutic agents' pharmacokinetic and pharmacodynamic potential.

## Definition

Hydrogen bonds form when a hydrogen atom covalently bonded to a strongly electronegative atom, such as nitrogen, oxygen, or fluorine (the donor), interacts electrostatically with another electronegative atom (the acceptor).
This type of interaction is crucial for dictating the three-dimensional structure of molecules, their ability to interact with biological targets, and their overall behavior in biological environments.

The dual nature of hydrogen bond acceptors and donors within a molecule significantly impacts its solubility, ability to traverse cell membranes, and how it binds to its target, ultimately influencing the drug's effectiveness and bioavailability.
As such, the balanced consideration of both acceptors and donors is paramount in drug design strategies.

Info

MolModa defines a hydrogen-bond donor as any N–H or O–H bond.
The number of hydrogen-bond acceptors is the total O or N atoms.

## Role and interpretation

CADD leverages sophisticated algorithms to analyze the hydrogen bonding potential of drug candidates, focusing on the balance between hydrogen bond acceptors and donors.
This analysis helps in screening compound libraries, optimizing lead compounds, and evaluating drug-likeness by incorporating several critical considerations:

- **Solubility and Permeability Prediction:** Software tools assess the number and distribution of hydrogen bond donors and acceptors to predict a compound’s aqueous solubility and ability to permeate lipid bilayers.
  A harmonious balance between donors and acceptors is often sought to ensure adequate solubility without compromising membrane permeability.
- **Binding Affinity Optimization:** The interaction between a drug's hydrogen bond donors and acceptors and its biological target can significantly affect binding affinity.
  CADD models utilize this information to modify chemical structures, enhancing efficacy and selectivity.
- **Drug-likeness Evaluation:** Guidelines such as the "Rule of 5" emphasize the importance of hydrogen bonding in determining drug-likeness, with specific criteria for the acceptable number of hydrogen bond donors and acceptors.
  This underscores these interactions' integrated role in predicting drug candidates' success.

Back to top


CC BY-SA 4.0 by Durrant Lab
