# Supplementary material for: MolModa: accessible and secure molecular docking in a web browser
Source: Nucleic Acids Res. 2024 May 23;52(W1):W498–506. doi: 10.1093/nar/gkae406 (PMC11223821; doi:10.1093/nar/gkae406)
Supplement: gkae406_Supplemental_Files [file gkae406_supplemental_files.zip › molmoda_2024-04-13_17-40-53.zip!/molmoda_2024-04-13_17-40-53/docs/structures/molprop/index.html]

Molecular properties - MolModa Documentation


 


Skip to content

MolModa Documentation

Molecular properties


Initializing search

durrantlab/molmoda


- Home
- Interface
- Structures
- Tutorials

MolModa Documentation

durrantlab/molmoda

- Home
- Interface
- Structures


  Structures
  - Loading
  - Molecular properties

    Molecular properties
    - logP
    - Molecular weight
    - Hydrogen bonding
    - Molar refractivity
    - SP3-hybridized carbons
    - Surface area
    - Topological polar surface area
  - Pockets
- Tutorials

# Molecular properties

This documentation introduces the computation of critical molecular properties, such as logP, surface area, and molecular weight, which are pivotal in computer-aided drug design (CADD).
The software facilitates the rapid and accurate prediction of these properties, thereby aiding researchers and professionals in identifying, optimizing, and developing potential drug candidates.

Back to top


CC BY-SA 4.0 by Durrant Lab
