# Supplementary material for: MolModa: accessible and secure molecular docking in a web browser
Source: Nucleic Acids Res. 2024 May 23;52(W1):W498–506. doi: 10.1093/nar/gkae406 (PMC11223821; doi:10.1093/nar/gkae406)
Supplement: gkae406_Supplemental_Files [file gkae406_supplemental_files.zip › molmoda_2024-04-13_17-40-53.zip!/molmoda_2024-04-13_17-40-53/docs/structures/molprop/mr/index.html]

Molar refractivity - MolModa Documentation


 


Skip to content

MolModa Documentation

Molar refractivity


Initializing search

durrantlab/molmoda


- Home
- Interface
- Structures
- Tutorials

MolModa Documentation

durrantlab/molmoda

- Home
- Interface
- Structures


  Structures
  - Loading
  - Molecular properties

    Molecular properties
    - logP
    - Molecular weight
    - Hydrogen bonding
    - Molar refractivity


      Molar refractivity


      Table of contents
      - Methodology

        - Atom classification
        - Calculation
    - SP3-hybridized carbons
    - Surface area
    - Topological polar surface area
  - Pockets
- Tutorials

Table of contents

- Methodology

  - Atom classification
  - Calculation

# Molar refractivity

The Wildman-Crippen molar refractivity (MR) is a physicochemical parameter that provides insights into compounds' molecular size and polarizability.
Molar refractivity, in particular, is used as a common descriptor for molecular size and polarizability, which are significant for predicting how a molecule interacts with biological targets and membranes.

## Methodology

MolModa uses the method defined by Wildman and Crippen to compute the molar refractivity.
This technique involves aggregating the contributions from each atom within a molecule to determine its overall MR value.
While MR is not strictly additive due to intramolecular interactions, these interactions can be accommodated by classifying atoms into distinct types based on their attached and neighboring atoms.

### Atom classification

The foundational step in computing MR involves an atom classification system.
Atoms are classified into different types according to their nearest neighbors, second neighbors, and aromaticity.
This process results in a reduced set of atom types, designed to include atoms with similar chemical natures and similar contributions to MR.
Ultimately, the classification system encompasses 68 basic atom types, covering elements commonly found in organic molecules (e.g., C, H, N, O, S, P, halogens), metals, and noble gases.

Atom Type Descriptions and Contributions

| type | descriptions | SMARTS | MR \(\alpha\_i\) |
| --- | --- | --- | --- |
| C1 | 1°, 2° aliphatic | `[CH4]`, `[CH3]C`, `[CH2](C)C` | 2.503 |
| C2 | 3°, 4° aliphatic | `[CH](C)(C)C`, `[C](C)(C)(C)C` | 2.433 |
| C3 | 1°, 2° | `[CH3][(N,O,P,S,F,Cl,Br,I)]`, `[CH3][A#N]`, `[CH2X4][A#N]`, `[CH3][#15]`, `[CH2X4][#15]`, `[CH3][#16]`, `[CH2X4][#16]`, `[CH3][#53]`, `[CH2X4][#53]`, `!([CH2X4]a)` | 2.753 |
|  | heteroatom | `[CH2X4][(N,O,P,S,F,Cl,Br,I)]` |  |
| C4 | 3°, 4° | `[CH1X4][(N,O,P,S,F,Cl,Br,I)]`, `[CHX4][A#N]`, `[CH0X4][A#N]`, `[CHX4][#15]`, `[CH0X4][#15]`, `[CHX4][#16]`, `[CH0X4][#16]`, `[CHX4][#53]`, `!([CH0X4][#53])`, `!([CHX4]a)` or `!([CH0X4]a)` | 2.731 |
|  | heteroatom | `[CH0X4][(N,O,P,S,F,Cl,Br,I)]` |  |
| C5 | C = heteroatom | `[C] = [A#X]` | 5.007 |
| C6 | C = C aliphatic | `[CH2] = C`, `[CH1](=C)A`, `[CH0](=C)(A)A`,`[C](=C)=C` | 3.513 |
| C7 | acetylene, nitrile | `[CX2]#A` | 3.888 |
| C8 | 1° aromatic carbon | `[CH3]c` | 2.464 |
| C9 | 1° aromatic heteroatom | `[CH3][a#X]` | 2.412 |
| C10 | 2° aromatic | `[CH2X4]a` | 2.488 |
| C11 | 3° aromatic | `[CHX4]a` | 2.582 |
| C12 | 4° aromatic | `[CH0X4]a` | 2.576 |
| C13 | aromatic heteroatom | `[cH0]−[!(C,N,O,S,F,Cl,Br,I)]`, `[c][#5]`, `[c][#14]`, `[c][#15]`, `[c][#33]`, `[c][#34]`, `[c][#50]`, `[c][#80]` | 4.041 |
| C14 | aromatic halide | `[c][#9]` | 3.257 |
| C15 | aromatic halide | `[c][#17]` | 3.564 |
| C16 | aromatic halide | `[c][#35]` | 3.180 |
| C17 | aromatic halide | `[c][#53]` | 3.104 |
| C18 | aromatic | `[cH]` | 3.350 |
| C19 | aromatic bridgehead | `[c](:a)(:a):a` | 4.346 |
| C20 | 4° aromatic | `[c](:a)(:a)-a` | 3.904 |
| C21 | 4° aromatic | `[c](:a)(:a)-C` | 3.509 |
| C22 | 4° aromatic | `[c](:a)(:a)-N` | 4.067 |
| C23 | 4° aromatic | `[c](:a)(:a)-O` | 3.853 |
| C24 | 4° aromatic | `[c](:a)(:a)-S` | 2.673 |
| C25 | 4° aromatic | `[c](:a)(:a) = C`, `[c](:a)(:a) = N`, `[c](:a)(:a) = O` | 3.135 |
| C26 | C = C aromatic | `[C](=C)(a)A`, `[C](=C)(c)a`, `[CH](= C)a`, `[C] = c` | 4.305 |
| C27 | aliphatic heteroatom | `[CX4][!(C,N,O,P,S,F,Cl,Br,I)]`, `[CX4][#X]`, `!([CX4][#N])`, `[CX4][#16]`, `[CX4][#15]`, `[CX4][#53]` | 2.693 |
| CS | carbon supplemental | `[#6]` not matching any basic C type | 3.243 |
| H1 | hydrocarbon | `[#1][#6]`, `[#1][#1]` | 1.057 |
| H2 | alcohol | `[#1]O[CX4]`, `[#1]Oc`, `[#1]O[!(C,N,O,S)]`, `[#1][!(C,N,O)]`, `[#1]O[CX4]`, `[#1]Oc`, `[#1]O[#1]`, `[#1]O[#5]`, `[#1]O[#14]`, `[#1]O[#15]`, `[#1]O[#33]`, `[#1]O[#50]`, `[#1][#5]`, `[#1][#14]`, `[#1][#15]`, `[#1][#16]`, `[#1][#50]` | 1.395 |
| H3 | amine | `[#1][#7]`, `[#1]O[#7]` | 0.9627 |
| H4 | acid | `[#1]OC = [#6]`, `[#1]OC = [#7]`, `[#1]OC = O`, `[#1]OC = S`, `[#1]OO`, `[#1]OS` | 1.805 |
| HS | hydrogen supplemental | `[#1]` not matching any basic H type | 1.112 |
| N1 | 1° amine | `[NH2+0]A` | 2.262 |
| N2 | 2° amine | `[NH+0](A)A` | 2.173 |
| N3 | 1° aromatic amine | `[NH2+0]a` | 2.827 |
| N4 | 2° aromatic amine | `[NH+0](A)a`, `[NH+0](a)a` | 3.000 |
| N5 | imine | `[NH+0] = A`, `[NH+0] = a` | 1.757 |
| N6 | substituted imine | `[N+0](=A)A`, `[N+0](=A)a`, `[N+0](=a)A`, `[N+0](=a)a` | 2.428 |
| N7 | 3° amine | `[N+0](A)(A)A` | 1.839 |
| N8 | 3° aromatic amine | `[N+0](a)(A)A`, `[N+0](a)(a)A`, `[N+0](a)(a)a` | 2.819 |
| N9 | nitrile | `[N+0]#A` | 1.725 |
| N10 | protonated amine | `[NH3+*]`, `[NH2+*]`, `[NH+*]` |  |
| N11 | unprotonated aromatic | `[n+0]` | 2.202 |
| N12 | protonated aromatic | `[n+*]` |  |
| N13 | 4° amine | `[NH0+*](A)(A)(A)A`, `[NH0+*](=A)(A)A`, `[NH0+*](=A)(A)a`, `[NH0+*](=[#6])=[#7]` | 0.2604 |
| N14 | other ionized nitrogen | `[N+*]#A`, `[N−*]`, `[N+*](=[N−*])=N` | 3.359 |
| NS | nitrogen supplemental | `[#7]` not matching any basic N type | 2.134 |
| O1 | aromatic | `[o]` | 1.080 |
| O2 | alcohol | `[OH]`, `[OH2]` | 0.8238 |
| O3 | aliphatic ether | `[O](C)C`, `[O](C)[A#X]`, `[O]([A#X])[A#X]` | 1.085 |
| O4 | aromatic ether | `[O](A)a`,`[O](a)a` | 1.182 |
| O5 | oxide | `[O]=[#8]`, `[O]=[#7]`, `[OX1−*][#7]` | 3.367 |
| O6 | oxide | `[OX1−*][#16]` | 0.7774 |
| O7 | oxide | `[OX1−*][!(N,S)]`, `[OX1−*][#15]`, `[OX1−*][#33]`, `[OX1−*][#43]`, `[OX1−*][#53]` | 0.000 |
| O8 | aromatic carbonyl | `[O]=c` | 3.135 |
| O9 | carbonyl aliphatic | `[O]=[CH]C`, `[O]=C(C)C`, `[O]=C(C)[A#X]`,`[O]=[CH]N`, `[O]=[CH]O`,`[O]=[CH2]`, `[O]=[CX2]=O` | 0.000 |
| O10 | carbonyl aromatic | `[O]=[CH]c`, `[O]=C(C)c`, `[O]=C(c)c`, `[O]=C(c)[a#X]`, `[O]=C(c)[A#X]`, `[O]=C(C)[a#X]` | 0.2215 |
| O11 | carbonyl heteroatom | `[O]=C([A#X])[A#X]`, `[O]=C([A#X])[a#X]`, `[O]=C([a#X])[a#X]` | 0.3890 |
| O12 | acid | `[O−1]C(=O)` |  |
| OS | oxygen supplemental | `[#8]` not matching any basic O type | 0.6865 |
| F | fluorine | `[#9−0]` | 1.108 |
| Cl | chlorine | `[#17−0]` | 5.853 |
| Br | bromine | `[#35−0]` | 8.927 |
| I | iodine | `[#53−0]` | 14.02 |
| Hal | ionic halogens | `[#9−*]`, `[#17−*]`, `[#35−*]`, `[#53−*]`, `[#53+*]` |  |
| P | phosphorous | `[#15]` | 6.920 |
| S1 | aliphatic | `[S−0]` | 7.591 |
| S2 | ionic sulfur | `[S−*]`, `[S+*]` | 7.365 |
| S3 | aromatic | `[s]` | 6.691 |
| Me1 | `B`, `Si`, `Ga`, `Ge`, `As`, `Se`, `Sn`, `Te`, `Pb`, `Ne`, `Ar`, `Kr`, `Xe`, `Rn` |  | 5.754 |
| Me2 | `Fe`, `Cu`, `Zn`, `Tc`, `Cd`, `Pt`, A`u,`Hg` |  |  |

These SMARTS strings are based on v1988.10 Molecular Operating Environment (MOE).

Reproduced from Wildman and Crippen [^wildman1999prediction].

### Calculation

Once the atoms in a molecule are classified, the MR is calculated as the sum of the contributions of each atom type present in the molecule. The formula for the calculation is as follows:

\[ \text{MR}\_{\text{calc}} = \sum\_{i} n\_{i} \alpha\_{i} \]

- \( n\_{i} \) represents the number of atoms of type \( i \) present in the molecule.
- \( \alpha\_{i} \) is the contribution to MR of atoms of type \( i \).

This equation is derived from the understanding that a molecule's MR can be approximated by summing the individual contributions of its constituent atoms, with each atom type having a unique contribution value based on its chemical environment.

Back to top


CC BY-SA 4.0 by Durrant Lab
