# Supplementary material for: MolModa: accessible and secure molecular docking in a web browser
Source: Nucleic Acids Res. 2024 May 23;52(W1):W498–506. doi: 10.1093/nar/gkae406 (PMC11223821; doi:10.1093/nar/gkae406)
Supplement: gkae406_Supplemental_Files [file gkae406_supplemental_files.zip › molmoda_2024-04-13_17-40-53.zip!/molmoda_2024-04-13_17-40-53/docs/structures/molprop/tpsa/index.html]

Topological polar surface area - MolModa Documentation


 


Skip to content

MolModa Documentation

Topological polar surface area


Initializing search

durrantlab/molmoda


- Home
- Interface
- Structures
- Tutorials

MolModa Documentation

durrantlab/molmoda

- Home
- Interface
- Structures


  Structures
  - Loading
  - Molecular properties

    Molecular properties
    - logP
    - Molecular weight
    - Hydrogen bonding
    - Molar refractivity
    - SP3-hybridized carbons
    - Surface area
    - Topological polar surface area
  - Pockets
- Tutorials

# Topological polar surface area

MolModa computes the polar surface area using the method described in Ertl, Rhote, and Selzer.

The process of calculating the Topological Polar Surface Area (TPSA) is streamlined into a series of steps, beginning with the identification of polar fragments within a molecule.
This identification is performed by analyzing the molecule's topological information, which can be represented in formats such as SMILES.
These topological descriptors allow for the recognition of various polar fragments, including those containing polar atoms such as oxygen, nitrogen, sulfur, and phosphorus, considering their specific bonding patterns.
Following the identification of these fragments, the next step involves the summation of their contributions to the molecule's overall polar surface area.
Each fragment type is associated with a predetermined surface area value, obtained from empirical data, which reflects its contribution to the total polar surface area of the molecule.

The mathematical formulation of this calculation is represented by the equation

\[
\text{TPSA} = \sum\_{i=1}^{n} c\_i \times n\_i,
\]

where

- \( c\_i \) denotes the surface area contribution of the \(i^{th}\) polar fragment type, and
- \( n\_i \) indicates the number of occurrences of this fragment within the molecule.

The sum is taken over all \( n \) distinct types of polar fragments identified in the molecule. This approach simplifies the traditional PSA calculation by leveraging the molecule's topological information, bypassing the need for computationally intensive 3D structure generation and direct surface area computation.

Back to top


CC BY-SA 4.0 by Durrant Lab
