# Supplementary material for: MolModa: accessible and secure molecular docking in a web browser
Source: Nucleic Acids Res. 2024 May 23;52(W1):W498–506. doi: 10.1093/nar/gkae406 (PMC11223821; doi:10.1093/nar/gkae406)
Supplement: gkae406_Supplemental_Files [file gkae406_supplemental_files.zip › molmoda_2024-04-13_17-40-53.zip!/molmoda_2024-04-13_17-40-53/docs/structures/molprop/surf/index.html]

Surface area - MolModa Documentation


 


Skip to content

MolModa Documentation

Surface area


Initializing search

durrantlab/molmoda


- Home
- Interface
- Structures
- Tutorials

MolModa Documentation

durrantlab/molmoda

- Home
- Interface
- Structures


  Structures
  - Loading
  - Molecular properties

    Molecular properties
    - logP
    - Molecular weight
    - Hydrogen bonding
    - Molar refractivity
    - SP3-hybridized carbons
    - Surface area


      Surface area


      Table of contents
      - Methodology
    - Topological polar surface area
  - Pockets
- Tutorials

Table of contents

- Methodology

# Surface area

In the context of computer-aided drug design, van der Waals surface area (VSA) is a key physicochemical property that influences molecular recognition, solubility, and permeability.
While there are no universal numerical guidelines for VSA that apply across all molecules or biological targets, specific general ranges and principles are considered when evaluating a compound's drug-like properties or its interaction with biological systems:

- **Drug Solubility and Permeability:** A larger VSA can indicate lower solubility, as the molecule has more surface area to interact with solvent molecules.
  Similarly, molecules with excessively large VSAs may face challenges in permeating cellular membranes, affecting their bioavailability.
  While specific cutoffs vary, a balance in VSA is crucial for optimal solubility and permeability.
- **Molecular Size and Drug-likeness:** The VSA is directly related to the size of the molecule.
  The Lipinski's Rule of Five suggests that molecules with a molecular weight less than 500 Dalton tend to have better drug-like properties, implying a limit on the VSA for orally active drugs.
  Although the rule does not directly mention VSA, molecules adhering to this guideline typically have a VSA that supports adequate solubility and permeability.
- **Optimal VSA for Target Binding:** The optimal VSA for binding to a biological target varies significantly across different targets.
  For instance, enzymes with deep, narrow active sites may favor smaller molecules with a smaller VSA.
  At the same time, interactions with broad, shallow surfaces, such as protein-protein interaction inhibitors, may accommodate larger VSAs.

## Methodology

MolModa computes the approximate surface area using Labute's method.

Each atom in a molecule is conceptualized as a sphere, with its radius equivalent to the van der Waals radius.
This simplification facilitates the computation of the VSA for each atom, which collectively contributes to the molecular VSA.
The calculation focuses on the portion of an atom's surface not overlapped by any other atom within the same molecule, thereby accounting for the exposed surface area crucial for molecular interactions.
The total VSA of a molecule is obtained by summing the VSAs of all constituent atoms.

Back to top


CC BY-SA 4.0 by Durrant Lab
