# Supplementary material for: MolModa: accessible and secure molecular docking in a web browser
Source: Nucleic Acids Res. 2024 May 23;52(W1):W498–506. doi: 10.1093/nar/gkae406 (PMC11223821; doi:10.1093/nar/gkae406)
Supplement: gkae406_Supplemental_Files [file gkae406_supplemental_files.zip › molmoda_2024-04-13_17-40-53.zip!/molmoda_2024-04-13_17-40-53/docs/structures/molprop/csp3/index.html]

SP3-hybridized carbons - MolModa Documentation


 


Skip to content

MolModa Documentation

SP3-hybridized carbons


Initializing search

durrantlab/molmoda


- Home
- Interface
- Structures
- Tutorials

MolModa Documentation

durrantlab/molmoda

- Home
- Interface
- Structures


  Structures
  - Loading
  - Molecular properties

    Molecular properties
    - logP
    - Molecular weight
    - Hydrogen bonding
    - Molar refractivity
    - SP3-hybridized carbons


      SP3-hybridized carbons


      Table of contents
      - Definition
      - Significance
      - Calculating SP3 fraction
    - Surface area
    - Topological polar surface area
  - Pockets
- Tutorials

Table of contents

- Definition
- Significance
- Calculating SP3 fraction

# SP3-hybridized carbons

SP3 hybridization plays a crucial role in chemistry, particularly in understanding the structure and reactivity of organic molecules.
This concept extends its importance into computer-aided drug design (CADD), which serves as a critical parameter for evaluating and designing potential drug candidates.
The percentage of carbon atoms that are SP3 hybridized (SP3 fraction) in a molecule is a key descriptor in medicinal chemistry, reflecting the three-dimensionality and complexity of molecular structures.

## Definition

SP3 hybridization occurs when one s orbital and three p orbitals in an atom mix to form four equivalent hybrid orbitals.
This configuration is characteristic of carbon atoms bonded in a tetrahedral geometry, prevalent in alkanes and other saturated organic compounds.
Molecules with a higher proportion of SP3-hybridized carbons tend to be more three-dimensional, potentially leading to more selective and efficacious drug interactions with biological targets.

## Significance

In computer-aided drug design, the SP3 fraction is used to evaluate the "drug-likeness" or "lead-likeness" of chemical compounds.
It is based on the principle that molecules with a higher SP3 fraction often exhibit better pharmacokinetic and pharmacodynamic profiles.
Such molecules are more likely to adopt diverse spatial conformations, enabling more specific interactions with the complex shapes of biological targets such as proteins and enzymes.

## Calculating SP3 fraction

The SP3 fraction of a molecule is calculated by dividing the number of SP3 hybridized carbon atoms by the total number of carbon atoms present in the molecule.
The formula can be represented as follows:

\[ \text{SP3 Fraction} = \frac{\text{Number of SP3 Hybridized Carbon Atoms}}{\text{Total Number of Carbon Atoms}} \]

Back to top


CC BY-SA 4.0 by Durrant Lab
