# Supplementary material for: MolModa: accessible and secure molecular docking in a web browser
Source: Nucleic Acids Res. 2024 May 23;52(W1):W498–506. doi: 10.1093/nar/gkae406 (PMC11223821; doi:10.1093/nar/gkae406)
Supplement: gkae406_Supplemental_Files [file gkae406_supplemental_files.zip › molmoda_2024-04-13_17-40-53.zip!/molmoda_2024-04-13_17-40-53/docs/structures/loading/index.html]

Loading - MolModa Documentation


 


Skip to content

MolModa Documentation

Loading


Initializing search

durrantlab/molmoda


- Home
- Interface
- Structures
- Tutorials

MolModa Documentation

durrantlab/molmoda

- Home
- Interface
- Structures


  Structures
  - Loading

    Loading
    - Proteins
    - Compounds
  - Molecular properties
  - Pockets
- Tutorials

# Loading structures

This documentation section shows how to load structural data into MolModa.
Proper preparation and loading of structural data are crucial for computational accuracy.
MolModa supports loading two types of structures:

- Proteins
- Compounds

Back to top


CC BY-SA 4.0 by Durrant Lab
