# Supplementary material for: MolModa: accessible and secure molecular docking in a web browser
Source: Nucleic Acids Res. 2024 May 23;52(W1):W498–506. doi: 10.1093/nar/gkae406 (PMC11223821; doi:10.1093/nar/gkae406)
Supplement: gkae406_Supplemental_Files [file gkae406_supplemental_files.zip › molmoda_2024-04-13_17-40-53.zip!/molmoda_2024-04-13_17-40-53/docs/structures/loading/proteins/index.html]

Proteins - MolModa Documentation


 


Skip to content

MolModa Documentation

Proteins


Initializing search

durrantlab/molmoda


- Home
- Interface
- Structures
- Tutorials

MolModa Documentation

durrantlab/molmoda

- Home
- Interface
- Structures


  Structures
  - Loading

    Loading
    - Proteins


      Proteins


      Table of contents
      - Protein Data Bank
      - AlphaFold
    - Compounds
  - Molecular properties
  - Pockets
- Tutorials

Table of contents

- Protein Data Bank
- AlphaFold

# Proteins

The `File` menu, shown below, contains several ways to import or load biomolecular into MolModa.


## Protein Data Bank

You can load structures directly from the Protein Data Bank (PDB) by clicking `File`  `PDB ID`.


For example, we can load in 1XDN, a RNA editing ligase 1 from *Trypanosoma brucei*.


MolModa will automatically load all chains; 8GTT for example, will have all seven chains shown by expanding `8GTT` in the navigator.


## AlphaFold

Not all structures are available in the PDB.
You can also load structures directly from the AlphaFold Protein Structure Database by clicking `File`  `AlphaFold`.


Let's go ahead and load the same RNA-editing ligase, but from the Alphafold Protein Structure Database.
We do this by providing the UniProt accession number, P86927.

Back to top


CC BY-SA 4.0 by Durrant Lab
