# Supplementary material for: MolModa: accessible and secure molecular docking in a web browser
Source: Nucleic Acids Res. 2024 May 23;52(W1):W498–506. doi: 10.1093/nar/gkae406 (PMC11223821; doi:10.1093/nar/gkae406)
Supplement: gkae406_Supplemental_Files [file gkae406_supplemental_files.zip › molmoda_2024-04-13_17-40-53.zip!/molmoda_2024-04-13_17-40-53/docs/structures/loading/compounds/index.html]

Compounds - MolModa Documentation


 


Skip to content

MolModa Documentation

Compounds


Initializing search

durrantlab/molmoda


- Home
- Interface
- Structures
- Tutorials

MolModa Documentation

durrantlab/molmoda

- Home
- Interface
- Structures


  Structures
  - Loading

    Loading
    - Proteins
    - Compounds


      Compounds


      Table of contents
      - PubChem
      - Text

        - Supported formats
      - Draw

        - How to Use the Drawing Tool
  - Molecular properties
  - Pockets
- Tutorials

Table of contents

- PubChem
- Text

  - Supported formats
- Draw

  - How to Use the Drawing Tool

# Compounds

The `File` menu, shown below, contains several ways to import or load compounds into MolModa.


## PubChem

Integrating molecular compounds into your drug design workflow is streamlined with our tool's ability to directly interface with PubChem, a robust chemical database.
This functionality enables you to easily import the structural data of compounds for docking experiments.
Below, we guide you through the process of loading compounds using different methods available in our tool.

PopupEnter molecule nameAuto CID lookup

To initiate the compound loading process, our tool offers a user-friendly pop-up interface that interacts with PubChem.
This method simplifies the selection and importation of compound structures directly from the database.
Through the pop-up interface, users can search for compounds by name, formula, or other identifiers.
The interface presents a list of matching results from PubChem, allowing for an intuitive selection process.

For users with specific compounds in mind, our tool provides an option to directly enter the name of the molecule.
This method bypasses the need for browsing the database manually, offering a quick and efficient way to proceed with the loading process.
Upon entering the molecule name, the system automatically queries PubChem for the corresponding compound. This feature is particularly useful for well-known compounds with unique names.

To further enhance user convenience, our tool incorporates an Auto CID lookup feature.
When a molecule name is provided, the tool not only searches for the compound but also automatically retrieves its Chemical Identifier (CID) from PubChem.
This automatic lookup streamlines the process, ensuring that the correct compound is selected without the need for manual verification of CIDs.

After selecting the compound through any of the aforementioned methods, the tool displays a confirmation of the loaded compound structure. This visual representation confirms that the correct compound is ready for further analysis and integration into your drug design workflow.


## Text

Our computer-aided drug design platform provides the flexibility to import molecular structures not only from databases like PubChem but also directly from external files or through pasting text-based representations.
This feature supports a variety of file formats and notation types, catering to the diverse needs of researchers in the field.


### Supported formats

The tool accepts molecular data in several widely used formats, ensuring compatibility with other software and databases in the field of drug discovery. Supported formats include:

- PDB (Protein Data Bank): A standard format for the three-dimensional structural data of proteins and nucleic acids.
- mol2: A file format used to describe the structure of chemical molecules containing atoms, bonds, coordinates, and other information.
- SDF (Structure-Data File): A file format used to represent multiple chemical compound information, supporting a wide range of chemical data fields.
- SMILES (Simplified Molecular Input Line Entry System): A notation that allows a user to represent a chemical structure in a way that can be used by the computer.

smilessdf

```
CS(=O)(=O)CCNCC1=CC=C(O1)C2=CC3=C(C=C2)N=CN=C3NC4=CC(=C(C=C4)OCC5=CC(=CC=C5)F)Cl
```

```
9549299
-OEChem-03062421093D

48 51  0     0  0  0  0  0  0999 V2000
-7.7265   -2.1388   -0.5627 F   0  0  0  0  0  0  0  0  0  0  0  0
-6.5520   -1.2263   -2.1413 F   0  0  0  0  0  0  0  0  0  0  0  0
-5.6635   -2.7526   -0.8726 F   0  0  0  0  0  0  0  0  0  0  0  0
    7.5757   -0.6332   -0.0294 O   0  0  0  0  0  0  0  0  0  0  0  0
    5.2336   -0.8701   -0.0887 N   0  0  0  0  0  0  0  0  0  0  0  0
    1.5493    2.2571   -0.4496 N   0  0  0  0  0  0  0  0  0  0  0  0
-3.0422    1.5350    0.6638 N   0  0  0  0  0  0  0  0  0  0  0  0
    0.5340    0.1541   -0.9145 N   0  0  0  0  0  0  0  0  0  0  0  0
-1.7810   -0.2099   -0.3532 N   0  0  0  0  0  0  0  0  0  0  0  0
    6.6942   -2.7968    0.1058 C   0  0  0  0  0  0  0  0  0  0  0  0
    5.9755   -3.4855    1.2123 C   0  0  0  0  0  0  0  0  0  0  0  0
    7.4689   -3.3439    1.2527 C   0  0  0  0  0  0  0  0  0  0  0  0
    6.5573   -1.3108   -0.0120 C   0  0  0  0  0  0  0  0  0  0  0  0
    4.7693    0.4406   -0.2038 C   0  0  0  0  0  0  0  0  0  0  0  0
    3.3987    0.6921   -0.2676 C   0  0  0  0  0  0  0  0  0  0  0  0
    5.6757    1.4995   -0.2558 C   0  0  0  0  0  0  0  0  0  0  0  0
    2.9289    2.0035   -0.3838 C   0  0  0  0  0  0  0  0  0  0  0  0
    5.2118    2.8098   -0.3717 C   0  0  0  0  0  0  0  0  0  0  0  0
    3.8412    3.0613   -0.4355 C   0  0  0  0  0  0  0  0  0  0  0  0
    0.4182    1.4049   -0.4220 C   0  0  0  0  0  0  0  0  0  0  0  0
-4.3134    0.9472    0.8311 C   0  0  0  0  0  0  0  0  0  0  0  0
-0.7476    1.9131    0.1135 C   0  0  0  0  0  0  0  0  0  0  0  0
-1.8328    1.0548    0.1252 C   0  0  0  0  0  0  0  0  0  0  0  0
-6.0093   -0.6133    0.1060 C   0  0  0  0  0  0  0  0  0  0  0  0
-4.7528   -0.0310   -0.0608 C   0  0  0  0  0  0  0  0  0  0  0  0
-5.1305    1.3429    1.8902 C   0  0  0  0  0  0  0  0  0  0  0  0
-6.8264   -0.2175    1.1648 C   0  0  0  0  0  0  0  0  0  0  0  0
-6.3869    0.7606    2.0570 C   0  0  0  0  0  0  0  0  0  0  0  0
-6.4796   -1.6587   -0.8476 C   0  0  0  0  0  0  0  0  0  0  0  0
-0.5878   -0.5881   -0.8497 C   0  0  0  0  0  0  0  0  0  0  0  0
    6.7853   -3.3218   -0.8391 H   0  0  0  0  0  0  0  0  0  0  0  0
    5.4032   -2.9033    1.9243 H   0  0  0  0  0  0  0  0  0  0  0  0
    5.5678   -4.4696    1.0148 H   0  0  0  0  0  0  0  0  0  0  0  0
    8.0635   -4.2335    1.0844 H   0  0  0  0  0  0  0  0  0  0  0  0
    7.8877   -2.6599    1.9807 H   0  0  0  0  0  0  0  0  0  0  0  0
    4.5111   -1.5864   -0.0647 H   0  0  0  0  0  0  0  0  0  0  0  0
    2.7301   -0.1577   -0.1951 H   0  0  0  0  0  0  0  0  0  0  0  0
    6.7498    1.3839   -0.2121 H   0  0  0  0  0  0  0  0  0  0  0  0
    5.9167    3.6352   -0.4124 H   0  0  0  0  0  0  0  0  0  0  0  0
    3.4947    4.0879   -0.5260 H   0  0  0  0  0  0  0  0  0  0  0  0
    1.3118    3.2421   -0.5278 H   0  0  0  0  0  0  0  0  0  0  0  0
-0.8090    2.9237    0.5017 H   0  0  0  0  0  0  0  0  0  0  0  0
-2.9149    2.3598    1.2457 H   0  0  0  0  0  0  0  0  0  0  0  0
-4.1556   -0.3340   -0.9154 H   0  0  0  0  0  0  0  0  0  0  0  0
-4.8004    2.1031    2.5936 H   0  0  0  0  0  0  0  0  0  0  0  0
-7.8081   -0.6593    1.3114 H   0  0  0  0  0  0  0  0  0  0  0  0
-7.0230    1.0686    2.8818 H   0  0  0  0  0  0  0  0  0  0  0  0
-0.5233   -1.5970   -1.2382 H   0  0  0  0  0  0  0  0  0  0  0  0
1 29  1  0  0  0  0
2 29  1  0  0  0  0
3 29  1  0  0  0  0
4 13  2  0  0  0  0
5 13  1  0  0  0  0
5 14  1  0  0  0  0
5 36  1  0  0  0  0
6 17  1  0  0  0  0
6 20  1  0  0  0  0
6 41  1  0  0  0  0
7 21  1  0  0  0  0
7 23  1  0  0  0  0
7 43  1  0  0  0  0
8 20  2  0  0  0  0
8 30  1  0  0  0  0
9 23  1  0  0  0  0
9 30  2  0  0  0  0
10 11  1  0  0  0  0
10 12  1  0  0  0  0
10 13  1  0  0  0  0
10 31  1  0  0  0  0
11 12  1  0  0  0  0
11 32  1  0  0  0  0
11 33  1  0  0  0  0
12 34  1  0  0  0  0
12 35  1  0  0  0  0
14 15  1  0  0  0  0
14 16  2  0  0  0  0
15 17  2  0  0  0  0
15 37  1  0  0  0  0
16 18  1  0  0  0  0
16 38  1  0  0  0  0
17 19  1  0  0  0  0
18 19  2  0  0  0  0
18 39  1  0  0  0  0
19 40  1  0  0  0  0
20 22  1  0  0  0  0
21 25  1  0  0  0  0
21 26  2  0  0  0  0
22 23  2  0  0  0  0
22 42  1  0  0  0  0
24 25  2  0  0  0  0
24 27  1  0  0  0  0
24 29  1  0  0  0  0
25 44  1  0  0  0  0
26 28  1  0  0  0  0
26 45  1  0  0  0  0
27 28  2  0  0  0  0
27 46  1  0  0  0  0
28 47  1  0  0  0  0
30 48  1  0  0  0  0
M  END
> <PUBCHEM_COMPOUND_CID>
9549299

> <PUBCHEM_CONFORMER_RMSD>
1

> <PUBCHEM_CONFORMER_DIVERSEORDER>
14
103
40
167
262
127
147
237
84
198
256
105
145
213
227
90
139
239
125
85
174
121
157
206
134
132
220
188
224
152
126
143
115
214
7
173
138
202
100
204
124
68
184
60
176
253
196
112
82
104
193
133
89
136
137
251
190
170
233
261
59
98
191
162
32
245
123
250
135
75
208
52
186
88
260
201
93
67
106
172
43
119
217
159
57
81
146
72
11
148
99
240
171
140
6
114
207
131
263
10
116
244
95
164
203
66
179
265
110
234
20
141
111
264
197
77
130
169
192
16
50
17
15
49
257
235
149
47
165
183
156
226
166
241
4
61
153
199
238
48
92
216
219
248
154
200
181
185
150
18
69
225
120
35
151
64
42
254
194
117
144
178
25
41
168
5
9
44
252
247
101
71
246
255
108
189
22
56
80
62
231
46
160
215
19
209
109
175
221
229
128
51
45
155
107
228
259
163
187
122
8
23
129
182
83
218
118
242
177
58
3
158
249
24
2
53
161
113
91
180
21
31
222
94
102
142
65
210
86
205
13
76
12
211
79
195
78
1
30
26
34
54
70
212
63
223
258
230
55
33
38
236
27
243
36
73
97
74
37
232
96
39
87
29
28

> <PUBCHEM_MMFF94_PARTIAL_CHARGES>
48
1 -0.34
10 -0.1
11 -0.2
12 -0.2
13 0.63
14 0.12
15 -0.15
16 -0.15
17 0.1
18 -0.15
19 -0.15
2 -0.34
20 0.41
21 0.1
22 -0.15
23 0.41
24 -0.14
25 -0.15
26 -0.15
27 -0.15
28 -0.15
29 1.16
3 -0.34
30 0.47
31 0.1
32 0.1
33 0.1
34 0.1
35 0.1
36 0.37
37 0.15
38 0.15
39 0.15
4 -0.57
40 0.15
41 0.4
42 0.15
43 0.4
44 0.15
45 0.15
46 0.15
47 0.15
48 0.15
5 -0.55
6 -0.6
7 -0.6
8 -0.62
9 -0.62

> <PUBCHEM_EFFECTIVE_ROTOR_COUNT>
8.6

> <PUBCHEM_PHARMACOPHORE_FEATURES>
10
1 4 acceptor
1 5 donor
1 6 donor
1 7 cation
1 7 donor
3 6 8 20 cation
3 8 9 30 cation
6 14 15 16 17 18 19 rings
6 21 24 25 26 27 28 rings
6 8 9 20 22 23 30 rings

> <PUBCHEM_HEAVY_ATOM_COUNT>
30

> <PUBCHEM_ATOM_DEF_STEREO_COUNT>
0

> <PUBCHEM_ATOM_UDEF_STEREO_COUNT>
0

> <PUBCHEM_BOND_DEF_STEREO_COUNT>
0

> <PUBCHEM_BOND_UDEF_STEREO_COUNT>
0

> <PUBCHEM_ISOTOPIC_ATOM_COUNT>
0

> <PUBCHEM_COMPONENT_COUNT>
1

> <PUBCHEM_CACTVS_TAUTO_COUNT>
18

> <PUBCHEM_CONFORMER_ID>
0091B5F30000000E

> <PUBCHEM_MMFF94_ENERGY>
104.1356

> <PUBCHEM_FEATURE_SELFOVERLAP>
50.882

> <PUBCHEM_SHAPE_FINGERPRINT>
12107183 9 18342183241212946304
12236239 1 18271530879002097409
13008946 170 18265333001093312268
13533116 47 18335706048727083224
13782708 43 17894630310397625948
14251764 18 18272937154426712124
15183329 4 18338236067520474373
15467298 65 18270673276770196654
1577012 14 18272941539235062217
16993089 31 17386303064200624955
1768 124 18343021056726850302
1979834 28 17560240198998601494
19841028 212 18335697260580573562
20028762 73 18342459214648254966
20165401 70 18410572886220721698
21130935 74 18413390964011885409
21267235 1 18338525145280583820
21315759 40 18272375273487429721
21585481 151 17987244326922041294
21585483 132 18336814360089364307
21682296 61 18187650171631417465
21987483 16 18127408071743676331
22002106 203 18262225622593389970
23081809 10 18343028830148261357
23522609 53 18125192469519742537
23569943 247 18261671459066695075
25242607 90 18267015069563660634
3504750 166 18341896238930291433
3663271 9 18059862787105074865
406291 66 18335700478175846466
4073 2 18191594266517978248
4516262 110 18118404175662541724
4616759 239 16343712006828315387
5104073 3 17917992806189255240
513202 73 18187363186322696213
531348 171 18408602540325466821
54039377 194 18190184491338868342
5718773 13 18335698308990001550
5874358 3 17845632842565014583
6299153 45 18341899597378016775
636783 161 18057027035212051447
9555976 147 17702954612764470937
96874 4 18412822482151532363
9962374 69 18334852832230641162

> <PUBCHEM_SHAPE_MULTIPOLES>
564.79
25.87
3.71
1.31
4.57
1.61
0.26
20.2
3.68
-7.66
0.23
2.63
0.4
-4.75

> <PUBCHEM_SHAPE_SELFOVERLAP>
1226.55

> <PUBCHEM_SHAPE_VOLUME>
307

> <PUBCHEM_COORDINATE_TYPE>
2
5
10

$$$$
```

## Draw

For a more hands-on approach to molecule design, our drug design platform includes a drawing tool that enables the detailed creation of small-molecule compounds.
This powerful feature allows for the visualization and manual construction of molecules, providing an interactive way to design and modify structures with precision.

The drawing tool presents a canvas within the interface, where users can draw the structure of a molecule using a variety of drawing elements and tools.


### How to Use the Drawing Tool

- **Select a tool:** Click on the desired tool from the toolbar to begin creating your molecule.
  Options include drawing single, double, or triple bonds, adding atoms, and more.
- **Draw the molecule:** Click and drag on the canvas to place atoms and bonds.
  The drawing interface is intuitive, allowing you to visualize the molecular structure as it is created.
- **Edit as needed:** Use the selection tool to adjust atoms and bonds, or to correct any part of the molecule.
  The eraser tool can remove specific parts of the structure, while the charge tool can add charges to atoms.
- **Confirm the structure:** Once the molecule is complete, review the structure to ensure it matches the intended design.

Back to top


CC BY-SA 4.0 by Durrant Lab
