# Supplementary material for: MolModa: accessible and secure molecular docking in a web browser
Source: Nucleic Acids Res. 2024 May 23;52(W1):W498–506. doi: 10.1093/nar/gkae406 (PMC11223821; doi:10.1093/nar/gkae406)
Supplement: gkae406_Supplemental_Files [file gkae406_supplemental_files.zip › molmoda_2024-04-13_17-40-53.zip!/molmoda_2024-04-13_17-40-53/docs/structures/pockets/index.html]

Pockets - MolModa Documentation


 


Skip to content

MolModa Documentation

Pockets


Initializing search

durrantlab/molmoda


- Home
- Interface
- Structures
- Tutorials

MolModa Documentation

durrantlab/molmoda

- Home
- Interface
- Structures


  Structures
  - Loading
  - Molecular properties
  - Pockets

    Pockets
    - Detection
    - Druggability
- Tutorials

# Pockets

In computer-aided drug design, the identification and characterization of protein pockets are pivotal for the discovery and development of new therapeutics.
A protein pocket is a concave surface region on a protein where small molecules, such as drugs, can bind.
These pockets are essential for the protein's biological function.
They often serve as active sites for enzymatic reactions or as binding sites for small molecules, including substrates, cofactors, and inhibitors.

Understanding and identifying these pockets are crucial for structure-based drug design (SBDD) approaches, which aim to design or identify compounds that can fit into these pockets with high specificity and affinity, thereby modulating the protein's function.
The process of drug discovery can be significantly expedited by using computational methods to predict potential binding sites on target proteins, thus focusing experimental efforts on the most promising candidates.

One of the challenges in SBDD is the accurate detection of these pockets, especially considering the dynamic nature of proteins, which can change conformation upon ligand binding, a phenomenon known as induced fit.
Before a potential drug molecule (ligand) can be docked into a protein's structure to predict how it binds and affects its function, it's imperative to identify these potential binding pockets.
Computationally detecting these pockets involves analyzing the protein's three-dimensional structure to locate cavities that could be binding sites.

Detecting protein pockets is not straightforward due to protein structures' complex and dynamic nature.
Proteins can exhibit significant flexibility, and their pockets may not always be evident without a bound ligand.
Moreover, a protein may have multiple potential binding sites.
These necessitating methods can accurately predict the most likely interaction sites with high specificity and affinity for a potential drug compound.

Back to top


CC BY-SA 4.0 by Durrant Lab
