# Supplementary material for: MolModa: accessible and secure molecular docking in a web browser
Source: Nucleic Acids Res. 2024 May 23;52(W1):W498–506. doi: 10.1093/nar/gkae406 (PMC11223821; doi:10.1093/nar/gkae406)
Supplement: gkae406_Supplemental_Files [file gkae406_supplemental_files.zip › molmoda_2024-04-13_17-40-53.zip!/molmoda_2024-04-13_17-40-53/docs/structures/pockets/druggability/index.html]

Druggability - MolModa Documentation


 


Skip to content

MolModa Documentation

Druggability


Initializing search

durrantlab/molmoda


- Home
- Interface
- Structures
- Tutorials

MolModa Documentation

durrantlab/molmoda

- Home
- Interface
- Structures


  Structures
  - Loading
  - Molecular properties
  - Pockets

    Pockets
    - Detection
    - Druggability
- Tutorials

# Druggability

Druggability is pivotal in guiding research and development efforts towards targets with a higher probability of successful therapeutic intervention.
Druggability is the likelihood that a particular protein or target site can bind with small, drug-like molecules with sufficient strength to modulate its biological function.
This concept, which adds a structural dimension to traditional target validation, is increasingly considered in the early stages of drug development to streamline efforts and resources towards targets with promising therapeutic potential.

fpocketweb includes a druggability score based on the approach detailed by Schmidtke and Barril.
Utilizing a large dataset of druggable and undruggable cavities alongside fpocket, the researchers trained a logistic model to predict druggability with high accuracy.
This method challenges the traditional oversimplification of druggable sites and incorporates the role of polar groups within these sites, recognizing their significant impact on ligand recognition.

Back to top


CC BY-SA 4.0 by Durrant Lab
