# Supplementary material for: MolModa: accessible and secure molecular docking in a web browser
Source: Nucleic Acids Res. 2024 May 23;52(W1):W498–506. doi: 10.1093/nar/gkae406 (PMC11223821; doi:10.1093/nar/gkae406)
Supplement: gkae406_Supplemental_Files [file gkae406_supplemental_files.zip › molmoda_2024-04-13_17-40-53.zip!/molmoda_2024-04-13_17-40-53/docs/structures/pockets/detection/index.html]

Detection - MolModa Documentation


 


Skip to content

MolModa Documentation

Detection


Initializing search

durrantlab/molmoda


- Home
- Interface
- Structures
- Tutorials

MolModa Documentation

durrantlab/molmoda

- Home
- Interface
- Structures


  Structures
  - Loading
  - Molecular properties
  - Pockets

    Pockets
    - Detection


      Detection


      Table of contents
      - Fpocket

        - Voronoi Tessellation and Alpha Sphere Detection
        - Clustering of Alpha Spheres
        - Characterization and Ranking of Pockets
    - Druggability
- Tutorials

Table of contents

- Fpocket

  - Voronoi Tessellation and Alpha Sphere Detection
  - Clustering of Alpha Spheres
  - Characterization and Ranking of Pockets

# Detection

MolModa uses fpocketweb to automatically detect and place boxes over each pocket.

## Fpocket

This WASM-compiled tool is based on fpocket.
Fpocket uses a sophisticated yet intuitive methodology to identify and analyze potential ligand-binding pockets within protein structures.
This open-source software leverages the principles of Voronoi tessellation and alpha spheres, integrated into a comprehensive framework capable of handling large-scale datasets efficiently.
At its core, Fpocket employs the concept of alpha spheres.
In this geometric notion, a sphere touches four atoms of a protein on its boundary without enclosing any atom within its volume.
These spheres are instrumental in delineating the voids and crevices on the protein surface, indicative of potential binding sites.

### Voronoi Tessellation and Alpha Sphere Detection

The initial step involves decomposing the protein's 3D space into Voronoi cells using the Qhull algorithm.
This decomposition facilitates the identification of alpha spheres.
Fpocket then filters these spheres based on size criteria to discard those too small (indicative of tight atomic packing) or too large (signifying solvent-exposed regions), focusing on those of intermediate sizes that represent potential binding pockets.

### Clustering of Alpha Spheres

Subsequently, Fpocket undertakes a clustering process where alpha spheres in proximity are grouped together, forming clusters that signify potential pockets.
This process involves several stages, from initial rough segmentation based on neighbor lists to more refined clustering leveraging multiple linkage criteria, ensuring the comprehensive detection of pocket regions.

### Characterization and Ranking of Pockets

Once identified, potential pockets are characterized based on various descriptors, such as size, hydrophobicity, and the types of atoms defining the pocket perimeter.
A scoring function, derived using Partial Least Squares (PLS) regression, ranks these pockets based on their potential to bind small, drug-like molecules.
This ranking not solely determines drugability but highlights pockets with favorable binding characteristics.

Back to top


CC BY-SA 4.0 by Durrant Lab
