# Supplementary material for: MolModa: accessible and secure molecular docking in a web browser
Source: Nucleic Acids Res. 2024 May 23;52(W1):W498–506. doi: 10.1093/nar/gkae406 (PMC11223821; doi:10.1093/nar/gkae406)
Supplement: gkae406_Supplemental_Files [file gkae406_supplemental_files.zip › molmoda_2024-04-13_17-40-53.zip!/molmoda_2024-04-13_17-40-53/docs/docking/tutorials/index.html]

Tutorials - MolModa Documentation


 


Skip to content

MolModa Documentation

Tutorials


Initializing search

durrantlab/molmoda


- Home
- Interface
- Structures
- Tutorials

MolModa Documentation

durrantlab/molmoda

- Home
- Interface
- Structures
- Tutorials

  Tutorials
  - TD001. Epidermal growth factor receptor inhibitors

# Tutorials

This section provides step-by-step guides for using our docking software effectively.
The tutorials cover a range of topics from basic setup and running your first docking simulation to analyzing results and optimizing docking parameters for accurate binding affinity predictions.
These tutorials are designed to be practical, with clear instructions and best practices to enhance your drug discovery projects.

Back to top


CC BY-SA 4.0 by Durrant Lab
