# Supplementary material for: MolModa: accessible and secure molecular docking in a web browser
Source: Nucleic Acids Res. 2024 May 23;52(W1):W498–506. doi: 10.1093/nar/gkae406 (PMC11223821; doi:10.1093/nar/gkae406)
Supplement: gkae406_Supplemental_Files [file gkae406_supplemental_files.zip › molmoda_2024-04-13_17-40-53.zip!/molmoda_2024-04-13_17-40-53/docs/docking/tutorials/td001/index.html]

TD001. Epidermal growth factor receptor inhibitors - MolModa Documentation


 


Skip to content

MolModa Documentation

TD001. Epidermal growth factor receptor inhibitors


Initializing search

durrantlab/molmoda


- Home
- Interface
- Structures
- Tutorials

MolModa Documentation

durrantlab/molmoda

- Home
- Interface
- Structures
- Tutorials

  Tutorials
  - TD001. Epidermal growth factor receptor inhibitors


    TD001. Epidermal growth factor receptor inhibitors


    Table of contents
    - Learning objectives
    - Protein

      - Load PDB
      - Remove water molecules
      - Protonation
    - Pockets
    - Compounds

      - Protonate
    - Docking

      - Exhaustiveness

        - 8
        - 32
      - Pose refinement
    - Conclusions

Table of contents

- Learning objectives
- Protein

  - Load PDB
  - Remove water molecules
  - Protonation
- Pockets
- Compounds

  - Protonate
- Docking

  - Exhaustiveness

    - 8
    - 32
  - Pose refinement
- Conclusions

# TD001. Epidermal growth factor receptor inhibitors

The Epidermal Growth Factor Receptor (EGFR) is crucial for cell growth and division, and its malfunction is implicated in many cancers.
Targeting EGFR with FDA-approved inhibitors offers a strategic approach to cancer therapy.
This MolModa tutorial demonstrates how to dock compounds into the EGFR structure.

Tip

You may download the final MolModa file and see the results of this tutorial.

## Learning objectives

At the end of this tutorial, you should be able to

- Load a structure from the Protein Data Bank;
- Process and prepare a protein for docking;
- Identify binding pockets;
- Load in multiple compounds from a single SMILES file;
- Protonate several compounds at a pH of 7.4;
- Dock multiple ligands into a single protein and analyze the results.

## Protein

### Load PDB

This tutorial guides you through loading the PDB structure 3W32 into MolModa.
The 3W32 file provides a detailed representation of the EGFR kinase domain, a key target in cancer therapy.
To load the structure, select `File`  `PDB ID`, then type in `3W32` and click `Load`.
MolModa will load in the PDB file as shown below.


### Remove water molecules

A key step in preparing for a protein-ligand docking simulation involves the removal of water molecules from the protein structure.
This preprocessing step is essential for several reasons.

Firstly, water molecules in the protein structure can introduce noise and computational complexity to the docking process.
Water molecules can occupy potential binding sites or interact with the ligand in ways not representative of the dry environment within which the docking simulations typically aim to approximate.
Secondly, excluding water molecules simplifies the computational model, reducing the number of variables that must be considered during the docking process.
This simplification can lead to a more focused and efficient exploration of the potential binding modes of the ligand to the protein.

Furthermore, removing water molecules allows for specialized scoring functions and algorithms designed for 'dry' docking scenarios, which may not account for the dynamic and complex nature of water-protein and water-ligand interactions.
However, while removing water molecules is a common practice, the decision to do so should be informed by the specific objectives of the simulation and the characteristics of the protein-ligand system under study.
In some cases, the inclusion of key water molecules, especially those known to play a critical role in the binding process or in maintaining protein structure, may be necessary for a more accurate representation of the biological system.

This tutorial will ignore all crystallographic water molecules from our structure.
We can ignore the water molecules in two ways: hide or delete.

1. Original2a. Hide waters2b. Delete waters3. Result

Here, we see the original structure with the red oxygen atoms from our water molecules.

Tip

X-ray crystallography cannot determine the positions of hydrogen atoms; thus, we only have lone oxygen atoms for our water molecules.

One option is to hide water molecules by clicking the eye next to `Solvent`.
In most calculations in MolModa, there is an option only to use visible or selected molecules.
Hiding water molecules is generally the recommended way to proceed since we never lose information.

The other option is to delete the water molecules by clicking the `x` next to `Solvent`.
This accomplishes the same results, except we cannot see the water molecules again unless we load in the PDB structure again.

The result of option 2a would look like this.

### Protonation

As mentioned previously, no hydrogen atoms exist in the original PDB structure.
Since these are crucial to describe intermolecular interactions accurately, we have to add these in.
MolModa provides an automated way to protonate the protein by selecting `Proteins`  `Protonation`.

1. Original2. Menu3. Popup4. Protonated

Here is what our protein looks like with the "Sticks" style.
We can see that there are no hydrogen atoms.

Here is where you can find the protein protonation menu.

This menu will pop up and give you options to select what you want to protonate.

After hitting `Protonate`, we can see that our structure is now protonated with hydrogen atoms.
Notice that MolModa creates a new group for our protonated protein instead of overwriting the original protein structure.

To clean up our `Navigation`, we will delete our unprotonated structure.

## Pockets

Selecting the appropriate binding pocket for docking molecules is critical in the protein-ligand docking process.
The identification and selection of the binding pocket begin with a comprehensive analysis of the protein structure to pinpoint potential sites that can accommodate the ligand.
This analysis often involves a combination of computational methods and biological insights.

Understanding the protein's function and the biological context within which it operates is critical to this selection process.
Knowledge of the protein's active sites, obtained from literature reviews, experimental data, and databases of protein structures, provides invaluable guidance.
For proteins with known ligands, these active sites serve as primary targets for docking.
A more exploratory approach is required in cases where the binding site is unknown or the protein may interact with multiple ligands.
Here, algorithms that predict potential binding sites based on geometric, energetic, and chemical criteria are beneficial.

Selecting the right pocket for docking also involves evaluating the compatibility of the pocket's characteristics with the ligand's physicochemical properties.
The pocket's shape, size, and charge distribution should complement those of the ligand to facilitate a stable and biologically relevant interaction. Additionally, the pocket's accessibility, flexibility, and the presence of critical amino acids that can form key interactions with the ligand are considered to ensure that the selected site can realistically accommodate the ligand under physiological conditions.

Once a potential binding pocket is identified, it may be refined and optimized through further computational analysis to enhance the accuracy of the docking simulation.
This refinement can include adjusting the pocket's dimensions, optimizing the alignment of pocket residues, and incorporating flexibility into the pocket structure to allow for a more dynamic interaction with the ligand.

For this tutorial, we will use the pocket containing the co-crystallized ligand.

1. Detect pockets2. Found pockets3. Pocket properties4. Selected pocket5. Modified pocket6. Remove compounds

Select `Proteins`  `Detect Pockets` to get to this option menu.

After hitting `Detect,` MolModa will detect potential binding pockets and show them as rectangular positions in space.
MolModa identified 21 potential pockets, but we want to select the one that best captures our ligand.

If you do not have a ligand you are trying to capture with your pocket, you can see pocket properties under the `Data` tab of the main window.
The higher the score and druggability, the more likely the pocket is to be an effective binding site for a ligand.

As shown below, we deleted all pockets that did not contain the ligand.

Upon closer inspection, the original pocket does not entirely capture the ligand.
This can cause issues once we dock, so we will manually change the pocket's origin and dimensions.

We will delete the original compounds now that we have our defined region.

Note

You may have noticed that sulfate ions were excluded in our region.
This is irrelevant to our docking procedure, so we also removed it.

## Compounds

We first need to load the compounds of interest to conduct the docking simulations.
The compounds we'll be working with can be imported from a SMILES file, which encodes each compound's structure in text format.
This file can be downloaded here. The steps below guide you through loading these compounds into MolModa for docking.

1. Open molecules2. Loaded molecules3. Example molecule

To begin, navigate to `File`  `Open` and select the SMILES file you've downloaded (`td001.smiles`).
This action prompts MolModa to load the molecular structures contained within the file.

Upon loading, the molecules from the SMILES file will appear in the `Navigation` pane. Each molecule is listed separately, allowing for individual selection and manipulation.

Here's an example of how a single molecule from the SMILES file is represented in MolModa.

### Protonate

Before docking, it's crucial to protonate the compounds at a physiological pH (7.4) to ensure their ionization states accurately reflect biological conditions.
MolModa facilitates this process through an automated protonation tool.

Note

You can skip this step if you load compounds with the desired protonation state.

1. Before2. Options3. After

Initially, the loaded molecules may lack hydrogen atoms for the specified pH.
We will protonate the molecules regardless to ensure consistency in our protocol.

Access the protonation options by selecting `Compounds`  `Protonation`.
Ensure that the pH is set to 7.4 to reflect physiological conditions.

After protonation, the compounds are updated to include hydrogen atoms, better representing their reactive form under physiological pH.

## Docking

With the protein pocket selected and compounds prepared, we move on to the docking stage.
Docking allows us to predict how these compounds fit into the target site on the protein, providing insights into their potential effectiveness as inhibitors.

1. Menu2. Docking options3. Running

Begin docking by navigating to `Docking`  `Compound`.
This opens the docking menu, where you can select your target pocket and the compounds to dock.

In the docking options, you can specify parameters such as the pocket, exhaustiveness of the search, and a limit on rotatable bonds.
A higher exhaustiveness value increases the thoroughness of the search but also requires more computational time.

Once you start the docking process, MolModa will display the progress on the `Jobs` tab.
This process can take some time, depending on the number of compounds and the exhaustiveness setting.

### Exhaustiveness

#### 8

Setting the exhaustiveness to 8 provides a balance between speed and thoroughness.
The default setting of `8` is generally sufficient for most screenings.

Tip

Docking all 11 compounds took around 2 minutes on an i9-12900K CPU.

1. Docked poses2. Scores3. Top hit

After docking, MolModa displays each compound's different poses within the binding pocket.
These poses are ranked based on their docking scores.

The docking scores are indicative of the binding affinity.
Lower scores typically suggest a stronger binding interaction.

The pose with the lowest docking score is considered the top hit, implying it is the most favorable binding orientation.

#### 32

Increasing the exhaustiveness to 32 provides a more comprehensive search, potentially uncovering better-fitting poses.

Tip

Docking all 11 compounds with this exhaustiveness took around 10 minutes on an i9-12900K CPU.

1. Scores2. Top hit

With a more exhaustive search, you might observe changes in the docking scores, indicating different binding affinities.
We see that the order changes slightly, but within 1.0 kcal/mol of the lowest score, we have the same molecules `2`, `5`, `7`, and `10` for both exhaustiveness.

The top hit at this exhaustiveness level may differ from the one at a lower level, reflecting a possibly more accurate prediction of the ligand binding.

### Pose refinement

After identifying promising poses, further refinement can be performed to improve accuracy.
This step adjusts the ligand's conformation within the pocket for a more precise fit.
We changed the exhaustiveness to `400` to demonstrate computational tradeoffs.

Tip

Docking this compound with this exhaustiveness took around 8 minutes on an i9-12900K CPU.

1. Pose refinement2. Scores3. Top poses

Refinement operations can adjust the ligand's orientation and conformation, seeking to optimize interactions with the pocket.

The refined docking scores provide a more accurate estimation of binding affinity after the ligand's pose is adjusted.

The top poses, after refinement, exhibit the best fit and strongest interactions with the binding pocket, according to the refined scores.
We see only some slight rotations between the two exhaustiveness parameters.

## Conclusions

This completes the docking section of the tutorial.
You gained practical experience in protein-ligand docking through these steps, from preparing the protein and ligands to executing the docking simulation and analyzing the results.

Back to top


CC BY-SA 4.0 by Durrant Lab
